# SARS-CoV-2 spike protein receptor-binding domain perturbates intracellular calcium homeostasis and impairs pulmonary vascular endothelial cells

**DOI:** 10.1038/s41392-023-01556-8

**Published:** 2023-07-14

**Authors:** Kai Yang, Shiyun Liu, Han Yan, Wenju Lu, Xiaoqian Shan, Haixia Chen, Changlei Bao, Huazhuo Feng, Jing Liao, Shuxin Liang, Lei Xu, Haiyang Tang, Jason X.-J. Yuan, Nanshan Zhong, Jian Wang

**Affiliations:** 1 State Key Laboratory of Respiratory Disease, National Clinical Research Center for Respiratory Disease, Guangdong Key Laboratory of Vascular Disease, Guangzhou Institute of Respiratory Health, The First Affiliated Hospital of Guangzhou Medical University, Guangzhou, Guangdong China; 2 Guangzhou National Laboratory, Guangzhou International Bio Island, Guangzhou, Guangdong China; 3 Department of Pulmonary and Critical Care Medicine, The Affiliated Hospital of Inner Mongolia Medical University, Hohhot, Inner Mongolia Autonomous Region China; 4 Department of Pathology, The Affiliated Cancer Hospital of Guangzhou Medical University, Guangzhou, Guangdong China; 5 School of Medicine, Southern University of Science and Technology, Shenzhen, Guangdong China; 6 Section of Physiology, Division of Pulmonary, Critical Care and Sleep Medicine, Department of Medicine, University of California San Diego, La Jolla, CA USA

**Keywords:** Cardiovascular diseases, Respiratory tract diseases

## Abstract

Exposure to the spike protein or receptor-binding domain (S-RBD) of SARS-CoV-2 significantly influences endothelial cells and induces pulmonary vascular endotheliopathy. In this study, angiotensin-converting enzyme 2 humanized inbred (hACE2 Tg) mice and cultured pulmonary vascular endothelial cells were used to investigate how spike protein/S-RBD impacts pulmonary vascular endothelium. Results show that S-RBD leads to acute-to-prolonged induction of the intracellular free calcium concentration ([Ca^2+^]_i_) via acute activation of TRPV4, and prolonged upregulation of mechanosensitive channel Piezo1 and store-operated calcium channel (SOCC) key component Orai1 in cultured human pulmonary arterial endothelial cells (PAECs). In mechanism, S-RBD interacts with ACE2 to induce formation of clusters involving Orai1, Piezo1 and TRPC1, facilitate the channel activation of Piezo1 and SOCC, and lead to elevated apoptosis. These effects are blocked by Kobophenol A, which inhibits the binding between S-RBD and ACE2, or intracellular calcium chelator, BAPTA-AM. Blockade of Piezo1 and SOCC by GsMTx4 effectively protects the S-RBD-induced pulmonary microvascular endothelial damage in hACE2 Tg mice via normalizing the elevated [Ca^2+^]_i_. Comparing to prototypic strain, Omicron variants (BA.5.2 and XBB) of S-RBD induces significantly less severe cell apoptosis. Transcriptomic analysis indicates that prototypic S-RBD confers more severe acute impacts than Delta or Lambda S-RBD. In summary, this study provides compelling evidence that S-RBD could induce persistent pulmonary vascular endothelial damage by binding to ACE2 and triggering [Ca^2+^]_i_ through upregulation of Piezo1 and Orai1. Targeted inhibition of ACE2-Piezo1/SOCC-[Ca^2+^]_i_ axis proves a powerful strategy to treat S-RBD-induced pulmonary vascular diseases.

## Introduction

Since the end of 2019, the COVID-19 pandemic caused by severe acute respiratory syndrome coronavirus 2 (SARS-CoV-2) infection brings huge burden to the world that lacks efficient treatments and therapies. SARS-CoV-2 infection induces severe endotheliopathy, characterized by substantial endothelial cell damage and microthrombus aggregation in the pulmonary vasculature.^1^ Moreover, widespread microthrombi and hypercoagulation are observed more often in population suffering from severe COVID-19, suggesting pulmonary endotheliopathy as a common COVID-19 complication. Pulmonary vascular endothelial cell damage, such as apoptosis and loss of tight junctions, together with orchestrated intravascular coagulopathy, are more often seen in patients with severe COVID-19,^2^ raising the possibility that early diagnosis of these syndromes could largely benefit these patients.^3–5^ However, whether the observed endotheliopathy in postmortem lungs is mediated through direct viral binding remains controversial.^6–11^ It has been reported that exposure to spike protein or receptor-binding domain (S-RBD) that facilitates binding to receptors located on the plasma membrane of host cells, such as angiotensin-converting enzyme 2 (ACE2), can directly impact the endothelium of pulmonary vasculature in both cultured cell models and experimental animal models.^12–16^ At molecular level, the progressive vascular endotheliopathy develops through mechanisms involving the downregulation of ACE2 and impairment of mitochondria,^14^ and activation of immune responses involving crosstalk between vascular endothelial cells and innate immune cells.^12^ These evidence strongly imply the specific spike protein/S-RBD-host interaction, without complete viral infection and the subsequently induced microenvironmental changes, could be sufficient to damage the pulmonary vascular endothelium and drive the endotheliopathy and endothelial dysfunction in COVID-19. However, related underlying mechanisms remain unclear and require further study.

Studies have reported on the pulmonary hypertension (PH) occurrence and development in population infected by SARS-CoV-2 or with COVID-19.^17–22^ Upon SARS-CoV-2 infection, the predicted PH prevalence is supposed to be 12%^23^ and 39%^24^ in patients of non-intensive care unit (ICU) and ICU population, respectively. These clinical findings suggest the association among SARS-CoV-2, developmental pulmonary vascular damage and the occurrence of PH. Pulmonary vasculopathy associated with COVID-19 shares many pathophysiological and pathobiological features with that seen in PH, including smooth muscle hypertrophy and remodeling, endothelial dysfunction and microvascular microthrombi. During acute infection, the increased microthrombi in lung vessels may predispose the progression of chronic thromboembolic PH. During prolonged infection, persistent pulmonary vascular damage and microthrombus accumulation could together drive prolonged pulmonary vascular inflammation and remodeling, indicating PH as a long COVID complication that deserves further evaluation and attention.^25,26^ Previous studies have addressed that endothelial damage play vital roles to initiate the pulmonary vascular remodeling and PH. Endothelial cell death leads to imbalance between cell apoptosis and proliferation, which emerges and promotes the establishment of lumen-occluding lesions.^27–30^ In addition, endothelial cell damage stimulates smooth muscle cell proliferation and medial/adventitial hypertrophy through paracrine mechanisms.^31^ Together, these mechanisms promote the pathogenesis of PH-associated pulmonary vascular remodeling, which are also the potential mechanisms underlying S-RBD-induced vascular damage.

Intracellular virus-related increase of calcium is widely linked to various types of viral infections. As reviewed by Chen *et al*., intracellular calcium is essential for the entire process of viral infection in host cells, including viral entry, gene replication, virion maturation and release.^32^ Viruses may regulate calcium channels and disturb intracellular calcium homeostasis to benefit their lifecycle while inducing morbidity in host cells. Therefore, intracellular calcium level and the calcium channel expression are critical aspects for assisting virus-host interaction and viral infection. Similar observations have been reported regarding SARS-CoV-2-induced infections. The spike protein enters host macrophages via hijacking cellular calcium regulatory network and interacting with the TRP subfamily V member 2 (TRPV2) under febrile conditions.^33^ However, the detailed mechanisms of action how spike protein/S-RBD-induced virus-host interaction affects intracellular calcium homeostasis, and whether such effects contribute to the progressive pulmonary vascular endothelial damage, warrants further investigation.

Based on the pre-existing knowledge, by using in vivo ACE2 humanized inbred mice (hACE2 Tg), in vitro cultured human and mice pulmonary vascular endothelial cells, this study aims to systematically determine roles of spike protein/S-RBD on pulmonary vascular endothelium and endothelial cells, also explore the underlying mechanisms how spike protein/S-RBD influences the intracellular calcium homeostasis and induces vascular endothelial cell damage. This study demonstrates for the first time that spike protein/S-RBD could affect the activation and expression of calcium channels such as Piezo1 and store-operated calcium channels (SOCC) via mechanisms depending on S-RBD-ACE2 interaction, presence of intracellular calcium, and formation of ACE2-calcium channel clusters. These mechanisms are suppressed by specific inhibitor against S-RBD-ACE2 interaction, intracellular calcium chelator or inhibitors against Piezo1 and SOCC. These data highlight the potential role and contribution of calcium channels in spike protein/S-RBD-induced pulmonary vascular dysfunction and endothelial cell damage. Furthermore, by using specific calcium channel inhibitors, our findings strongly indicate that targeted inhibition of the intracellular calcium regulation could be a powerful strategy to treat the pulmonary vascular damage induced by S-RBD, providing novel insights into the treatment of COVID-associated pulmonary vascular diseases and management for long COVID complications.

## Results

### Acute exposure to receptor-binding domain (S-RBD) enhances intracellular calcium concentration ([Ca^2+^]_i_) via triggering TRPV4 activity in human pulmonary arterial endothelial cells (PAECs)

**Fig. 1:**
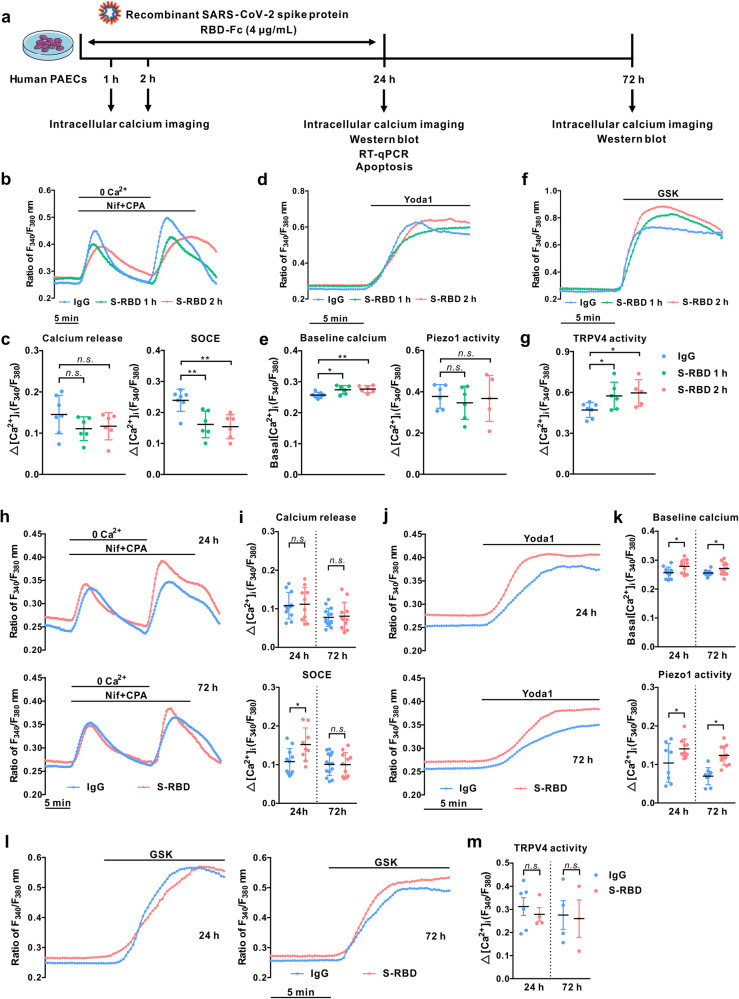
SARS-CoV-2 spike protein receptor-binding domain (S-RBD) induces an acute-to-prolonged perturbation of the intracellular calcium homeostasis in human pulmonary arterial endothelial cells (PAECs). **a** Schematic representation of the in vitro cell experimental design. **b**–**g** Representative traces and summarized data showing the effects of S-RBD (4 μg/mL for 1- and 2-hour) on intracellular calcium release induced by cyclopiazonic acid (CPA, 10 μM) and CPA-evoked store-operated calcium entry (SOCE) (**b**, **c**), baseline calcium and Piezo1 activity induced by agonist Yoda1 (0.5 μM) (**d**, **e**), TRPV4 activity induced by agonist GSK1016790A (GSK, 10 nM) (**f**, **g**) in cultured human PAECs. Bar values are mean ± SD, *n* = 5–7 experiments in 251 (IgG control, IgG), 213 (S-RBD 1 h) and 215 (S-RBD 2 h) cells for CPA-induced calcium release and SOCE, Yoda1-induced calcium response and baseline calcium measurements. Bar values are mean ± SD, *n* = 5–7 experiments in 269 (IgG control, IgG), 243 (S-RBD 1 h) and 229 (S-RBD 2 h) cells for GSK-induced calcium response. ^*^P < 0.05, ^**^P < 0.01 as indicated. **h**–**m** Representative traces and summarized data showing the effects of S-RBD (4 μg/mL for 24- and 72-hour) on intracellular calcium release induced by CPA (10 μM) and CPA-evoked SOCE (**h**, **i**), baseline calcium and Yoda1 (0.5 μM)-induced calcium increase (**j**, **k**), and GSK (10 nM)-induced calcium increase (**l**, **m**) in cultured human PAECs. Bar values are mean ± SD, *n* = 9–14 experiments in 344 (IgG 24 h), 401 (IgG 72 h), 321 (S-RBD 24 h) and 402 (S-RBD 72 h) cells for calcium release and SOCE measurements. Bar values are mean ± SD, *n* = 8–13 experiments in 357 (IgG 24 h), 322 (IgG 72 h), 341 (S-RBD 24 h) and 419 (S-RBD 72 h) cells for Yoda1 and baseline calcium measurements. Bar values are mean ± SD, *n* = 3-6 experiments in 240 (IgG 24 h), 169 (IgG 72 h), 158 (S-RBD 24 h) and 145 (S-RBD 72 h) cells for GSK measurements. ^*^P < 0.05 as indicated

The effects of S-RBD on intracellular calcium homeostasis in cultured human PAECs were investigated through acute exposure (1- and 2-hour) and prolonged exposure (24- and 72-hour) to S-RBD (Fig. 1a). We measured the activity of mechanosensitive channels Piezo1 and TRPV4, as well as SOCC, mainly constituted by STIM1, TRPC1 and Orai1.^34^ According to the dose-dependent experiments, 4 μg/mL S-RBD was chosen for subsequent molecular and functional assessments (Supplementary Fig. 1). Our results show that acute exposure (1- and 2-hour) to S-RBD leads to significant increases in baseline calcium (Fig. 1d, e) and TRPV4-mediated extracellular calcium influx induced by TRPV4 agonist GSK1016790A (GSK, 10 nM) (Fig. 1f, g), without affecting intracellular calcium release from internal calcium stores induced by cyclopiazonic acid (CPA, 10 μM), an specific blocker for SERCA (Fig. 1b, c), and Piezo1-mediated extracellular calcium influx induced by Piezo1 agonist Yoda1 (0.5 μM) (Fig. 1d, e). Acute exposure to S-RBD decreases the CPA-evoked store-operated calcium entry (SOCE) mediated by SOCC (Fig. 1b, c).

### Prolonged exposure to S-RBD elevates [Ca^2+^]_i_ via enhancing Piezo1 and SOCC activity in human PAECs

Next, we determined the effects of prolonged exposure to S-RBD (24- and 72-hour) on intracellular calcium homeostasis and observed different mechanisms compared to acute exposure. As shown in Fig. 1h–m, prolonged exposure to S-RBD for 24- and 72-hour both induce a significant elevation in baseline calcium levels and Yoda1-induced calcium increase through Piezo1 (Fig. 1j, k), but not CPA-induced intracellular calcium release (Fig. 1h, i) or GSK-induced calcium influx via TRPV4 (Fig. 1l, m). S-RBD exposure for 24-hours significantly increases CPA-evoked SOCE (Fig. 1h, i). The data presented in Fig. 1 suggests an acute-to-prolonged perturbation of intracellular calcium homeostasis in human PAECs induced by S-RBD exposure. Exposure to different spike protein subunits, such as S1 and S1 + S2, induces similar effects on elevated calcium responses (SOCC and Piezo1) and cell apoptosis (Supplementary Fig. 2).

### Prolonged exposure to S-RBD induces upregulation of Piezo1 and Orai1 in human PAECs

**Fig. 2:**
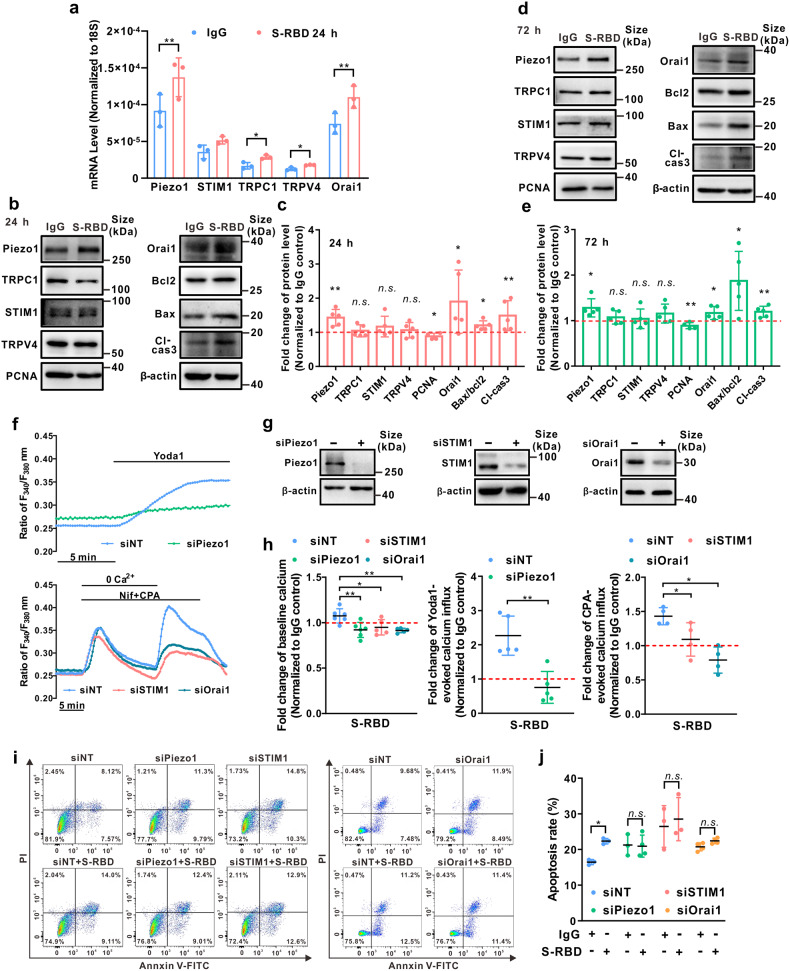
Upregulation of Piezo1 and Orai1 mediates the elevated intracellular calcium and apoptosis induced by prolonged exposure to S-RBD in human PAECs. **a** Bar graph showing the mRNA expression of TRPC1, STIM1, Orai1, TRPV4 and Piezo1 in cultured human PAECs treated with IgG or S-RBD for 24-hour. Bar values are mean ± SD, *n* = 3 experiments in each group. ^*^P < 0.05, ^**^P < 0.01 as indicated. **b**–**e** Representing the immunoblots (**b**, **d**) and analyzed bar graphs (**c**, **e**) showing the expression of TRPC1, STIM1, Orai1, TRPV4, Piezo1, Cleaved-Caspase 3, PCNA, Bax, Bcl2 and β-actin in cultured human PAECs treated with IgG or S-RBD for 24- and 72-hour. Bar values are mean ± SD, *n* = 5 experiments in each group. ^*^P < 0.05, ^**^P < 0.01 as indicated. **f**–**h** Representing the effects of specific siRNA knockdown of Piezo1, Orai1 or STIM1 on Yoda1-induced calcium increase, CPA-evoked SOCE and baseline calcium (**f**), protein expression of Piezo1, Orai1 or STIM1 (**g**), and effects on S-RBD-elevated baseline calcium, Piezo1 activity and SOCE (**h**) in human PAECs treated with IgG or S-RBD for 24-hour. Bar values are mean ± SD, *n* = 4–6 experiments in 204 (siNT + IgG), 233 (siNT + S-RBD), 160 (siPiezo1 + IgG), 245 (siPiezo1 + S-RBD), 168 (siSTIM1 + IgG), 226 (siSTIM1 + S-RBD), 148 (siOrai1 + IgG) and 116 (siOrai1 + S-RBD) cells. ^*^P < 0.05, ^**^P < 0.01 as indicated. **i**, **j** Representing flow cytometry (**i**) and graph (**j**) showing the effects of S-RBD (24-hour) and/or siSTIM1, siOrai1 or siPiezo1 on cell apoptosis in human PAECs. Bar values are mean ± SD, *n* = 3–4 experiments in each group. ^*^P < 0.05 as indicated

We then detected the potential effects of S-RBD on the mRNA and protein expression of related calcium channel components, including Piezo1, TRPV4, STIM1, TRPC1 and Orai1. As seen in Fig. 2, a 24-hour exposure to S-RBD significantly upregulates the mRNA expression of Piezo1, TRPC1, TRPV4 and Orai1, but not STIM1 (Fig. 2a). Prolonged exposure (24- and 72-hour) to S-RBD significantly upregulates the protein expression of Piezo1 and Orai1, in association with upregulated apoptotic markers (cleaved-caspase 3 and the ratio of Bax/Bcl2), and downregulates the proliferative marker PCNA, without affecting the expression of TRPC1, TRPV4, and STIM1 (Fig. 2b–e).

To evaluate whether the S-RBD-elevated [Ca^2+^]_i_ was due to the enhanced activity of Piezo1 and SOCC, we applied specific siRNA knockdown strategies against Piezo1 and two key SOCC components Orai1 and STIM1, the knockdown of which disables SOCC activity.^35,36^ Effective knockdown of Piezo1 (Fig. 2f, g) suppresses S-RBD-enhanced Piezo1 activity induced by Yoda1 and baseline calcium (Fig. 2h), whereas knockdown of either Orai1 or STIM1 (Fig. 2f, g) abolishes S-RBD-enhanced SOCE evoked by CPA and baseline calcium (Fig. 2h). Moreover, a 24-hour exposure to S-RBD induces a significant increase in cell apoptosis in human PAECs compared to the IgG treatment control, which could also be attenuated by the knockdown of Piezo1, Orai1, or STIM1 (Fig. 2i, j).

### S-RBD perturbates intracellular calcium homeostasis and induces cell apoptosis through interaction with ACE2 and formation of ACE2-bounded calcium channel clusters

**Fig. 3:**
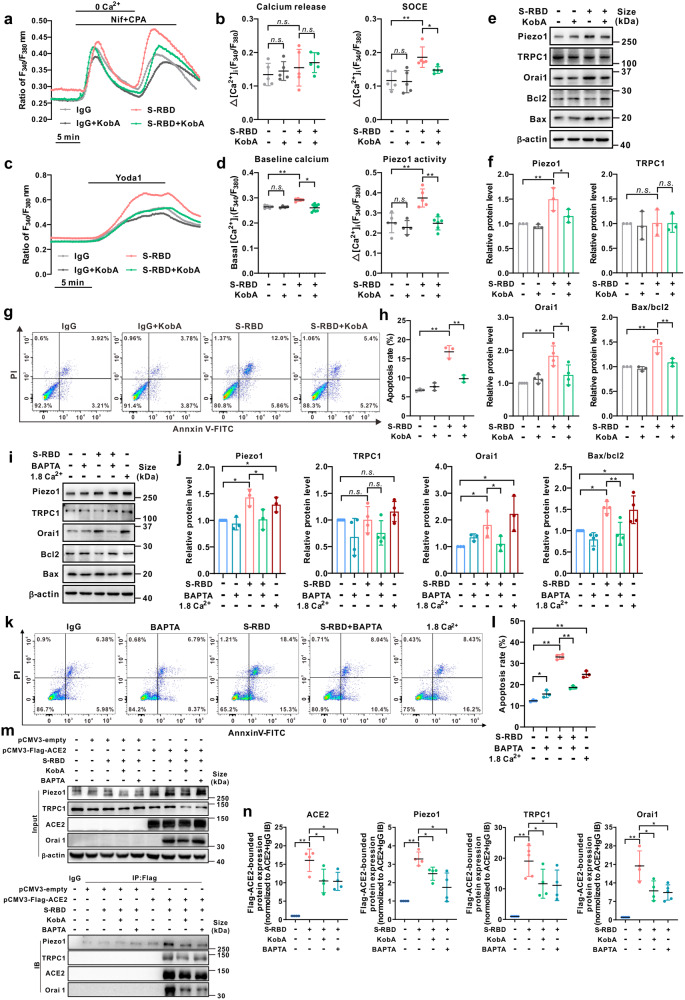
S-RBD induces perturbation of the intracellular calcium homeostasis and cell apoptosis through interaction with ACE2 and formation of ACE2-calcium channel clusters. **a**–**d** Representative traces and summarized data showing the effects of S-RBD (4 μg/mL for 24-hour) on intracellular calcium release induced by CPA (10 μM) and CPA-evoked SOCE (**a**, **b**), baseline calcium and Yoda1 (0.5 μM)-induced calcium increase (**c**, **d**) in cultured human PAECs. Bar values are mean ± SD, *n* = 4–6 experiments in each treatment group. *P < 0.05, **P < 0.01 as indicated. **e**, **f** Representing the immunoblots (**e**) and analyzed bar graphs (**f**) showing the expression of TRPC1, Orai1, Piezo1, Bax, Bcl2 and β-actin in cultured human PAECs treated with IgG or S-RBD, with or without KobA (10 μM) for 24-hour. Bar values are mean ± SD, *n* = 3 experiments in each group. *P < 0.05, **P < 0.01 as indicated. **g**, **h** Representing flow cytometry (**g**) and graph (**h**) showing the effects of S-RBD (24-hour) and/or KobA on cell apoptosis in human PAECs. Bar values are mean ± SD, *n* = 3 experiments in each group. **P < 0.01 as indicated. **i**, **j** Representing the immunoblots (**i**) and analyzed bar graphs (**j**) showing the expression of TRPC1, Orai1, Piezo1, Bax, Bcl2 and β-actin in cultured human PAECs treated with IgG or S-RBD, with or without BAPTA-AM (1 μM) for 24-hour. Bar values are mean ± SD, *n* = 3–4 experiments in each group. *P < 0.05, **P < 0.01 as indicated. **k**, **l** Representing flow cytometry (**k**) and graph (**l**) showing the effects of S-RBD (24-hour) and/or BAPTA-AM on cell apoptosis in human PAECs. Bar values are mean ± SD, *n* = 3–4 experiments in each group. **P < 0.01 as indicated. **m**, **n** Representing Co-IP (**m**) and analyzed bar graphs (**n**) showing the expression of TRPC1, Orai1, Piezo1 and ACE2 in Flag-ACE2-bounded fractions from pCMV3-Flag-ACE2-transfected HEK293T treated with IgG or S-RBD, with or without KobA (10 μM) or BAPTA-AM (1 μM) for 24-hour. Bar values are mean ± SD, *n* = 4–5 experiments in each group. *P < 0.05, **P < 0.01 as indicated

To determine whether S-RBD-induced apoptosis and calcium regulation are caused by the S-RBD-ACE2 interaction, we used the specific inhibitor Kobophenol A (KobA), which functionally blocks the interaction between ACE2 and S-RBD.^37^ As seen in Fig. 3, treatment with KobA (10 μM, 24-hour) significantly attenuates the S-RBD-induced activation of SOCC (Fig. 3a, b), Piezo1 and baseline calcium (Fig. 3c, d), upregulation of Orai1, Piezo1 and Bax/Bcl2 (Fig. 3e, f), and increased PAEC apoptosis (Fig. 3g, h), suggesting the importance and dependence of S-RBD-ACE2 interaction in S-RBD-induced calcium regulation and apoptosis. Interestingly, treatment with the intracellular calcium chelator BAPTA-AM (1 μM, 24-hour), which abolishes intracellular calcium ions,^38^ can also attenuate the S-RBD-induced upregulation of Orai1, Piezo1 and Bax/Bcl2 (Fig. 3i, j) and PAEC apoptosis (Fig. 3k, l), while treatment with extracellular calcium (1.8 mM CaCl_2_, 24-hour) significantly upregulates the protein expression of Orai1, Piezo1 and Bax/Bcl2 (Fig. 3i, j), and triggers cell apoptosis (Fig. 3k, l) in human PAECs. These data imply that elevated intracellular calcium levels are necessary to regulate the expression of calcium channel proteins (Piezo1 and Orai1) and cell apoptosis.

To further investigate the interplay between S-RBD-ACE2 interaction and calcium channel activation under S-RBD treatment or control conditions, HEK293T cells were transfected with pCMV3-Flag-ACE2, which encodes full-length human ACE2, or the control vector pCMV3-SP-N-FLAG-NCV (pCMV3-empty), before being treated with IgG or S-RBD for 24-hours. In ACE2-overexpressed cells, data from the co-immunoprecipitation (Co-IP) assay indicates that on the one hand, a 24-hour treatment with S-RBD induces remarkable upregulation of Piezo1 and Orai1, and on the other hand, S-RBD treatment significantly enhances the levels of Flag-ACE2-bounded Orai1, Piezo1, TRPC1, and ACE2. This suggests that S-RBD treatment may trigger the formation of clusters involving ACE2, Piezo1, Orai1 and TRPC1 that enable the open of Piezo1 and SOCC. Moreover, both KobA (10 μM) and BAPTA-AM (1 μM) can substantially attenuate the levels of Flag-ACE2-bounded Orai1, Piezo1, TRPC1 and ACE2, suggesting the S-RBD-triggered formation of ACE2-calcium channel clusters depends on the S-RBD-ACE2 interaction and presence of intracellular calcium (Fig. 3m, n). The activation of SOCC or calcium release-activated calcium channels (CRAC) depends on STIM1 activation and translocation upon internal calcium store depletion, where it binds to classical SOCC/CRAC components to initiate the opening of these channels.^39–41^ In Flag-ACE2-overexpressed HEK293T cells, although STIM1 protein expression is not altered by ACE2 overexpression and/or S-RBD treatment, S-RBD significantly enhances Flag-ACE2 binding with STIM1, indicating that STIM1 also participates in S-RBD-induced cluster formation and its downstream functional consequences (Supplementary Fig. 3). According to a previous report, internal calcium store depletion/STIM1-dependent reconstitution and activation of SOCC/CRAC (Orai/TRPC) proceed with relatively slow kinetics^42^; however, we attempted direct exposure to S-RBD (4 μg/mL mixed in KRB buffered solution) but failed to induce transient intracellular calcium responses in human PAECs (data not shown), suggesting that exposure to S-RBD could not directly induce SOCC/CRAC clustering and channel activation. Taken together, these data indicate that S-RBD-induced SOCC/CRAC activity relies on internal calcium depletion and STIM1 activation through relatively slow kinetics. During this process, upregulated Orai1 may contribute to an increased number of functional SOCC/CRAC channels.

### GsMTx4 inhibits the endothelial damage and pulmonary vascular remodeling in hACE2 Tg mice induced by S-RBD

**Fig. 4:**
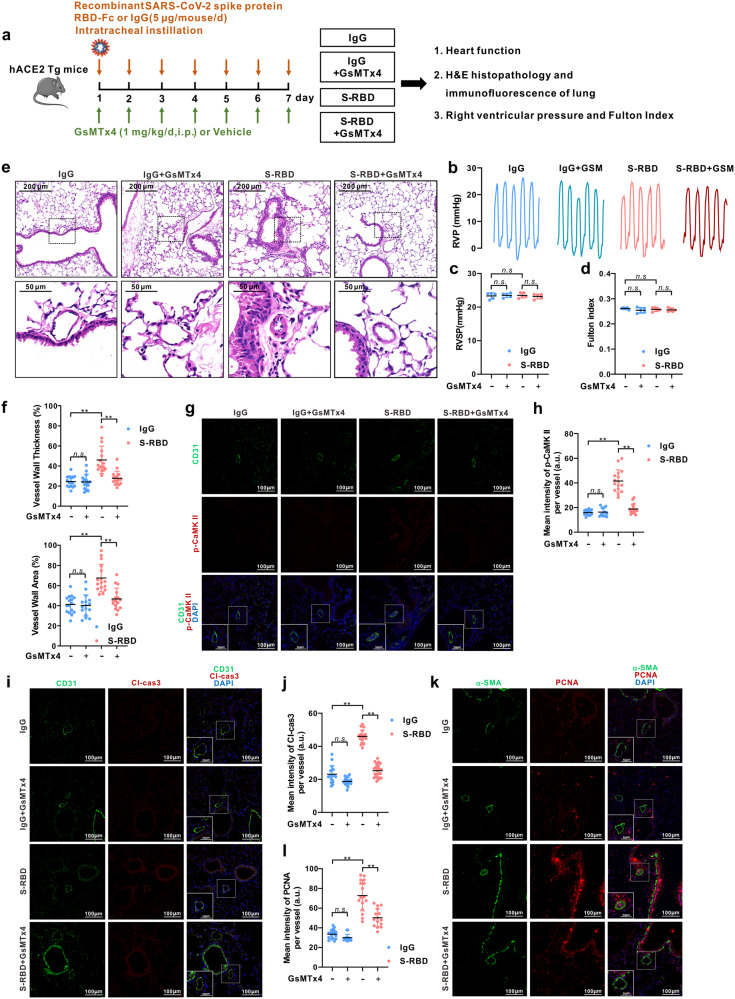
Effects of S-RBD and GsMTx4 on the hemodynamic changes and pulmonary vascular remodeling in hACE2 Tg mice. **a** Schematic representation of the in vivo animal experimental design. **b**–**d** Representing trace of right ventricular pressure (RVP, **b**), analyzed right ventricular systolic pressure (RVSP, **c**) and Fulton Index (**d**) in mice treated with S-RBD and/or GsMTx4 for 7-day. Graph values are mean ± SD, *n* = 5 mice in each group; **e**, **f** Representing H&E staining (**e**) and analyzed bar graphs (**f**) showing the % vessel wall thickness and area of pulmonary vasculature in mice treated with S-RBD and/or GsMTx4 for 7-day. Graph values are mean ± SD, *n* = 15–20 vessels from 5 mice in each group. **P < 0.01 as indicated. **g**–**l** Representing immunofluorescence dual staining labeling CD31 and p-CaMKII (**g**), CD31 and Cleaved-Caspase 3 (**i**), α-SMA and PCNA (**k**), as well as analyzed bar graphs (**h**, **j**, **l**) showing immunofluorescent intensity in the pulmonary vasculature of hACE2 Tg mice treated with S-RBD and/or GsMTx4 for 7-day. Graph values are mean ± SD, *n* = 15–25 vessels from 5 mice in each group. **P < 0.01 as indicated

The role of S-RBD on pulmonary vascular endothelium was assessed by an in vivo study using a 7-day intratracheal instillation of S-RBD or IgG, with or without GsMTx4, in hACE2 Tg mice expressing humanized ACE2 that enables binding to S-RBD,^43^ following the protocols shown in Fig. 4a. The comparative effects of the two control treatments (IgG and Fc-IgG) were first tested in hACE2 Tg mice and cultured human PAECs, which exhibit no significant differences in vivo or in vitro (Supplementary Fig. 4). Although typical characteristics of PH, such as the right ventricular systolic pressure (RVSP, Fig. 4b, c) and right ventricular hypertrophy indicated by Fulton Index (Fig. 4d), are not significantly altered by S-RBD and/or GsMTx4 treatment, histological analysis reveals that S-RBD induces remarkable pulmonary vascular thickening and remodeling in small to medial (with an outer diameter between 25 and 50 μm) pulmonary vessels, which are prevented by GsMTx4 treatment (Fig. 4e, f).

We also observed significantly elevated intracellular calcium levels and apoptosis rates in the pulmonary vascular endothelium upon treatment with S-RBD, reflected by increased phosphorylated calcium-dependent kinase (p-CaMKII) (Fig. 4g, h) and increased cleaved-caspase 3 immunoreactivity (Fig. 4i, j) levels in the specific endothelial cell marker CD31 positive region of pulmonary vessels. In addition, treatment with S-RBD also induces a significant proliferation of smooth muscle cells, indexed by elevated PCNA immunoreactivity in the α-SMA positive layer of smooth muscle cells (Fig. 4k, l). All changes are normalized in the GsMTx4 treatment group. GsMTx4 did not affect the pulmonary vasculature in IgG-treated hACE2 Tg mice.

Notably, neither S-RBD nor GsMTx4 lead to significant alterations in heart function, as reflected by the echocardiographic analysis. Among the assessed indices, only RV fractional area change (FAC) shows a significant decrease under S-RBD treatment and restoration upon GsMTx4 treatment (Supplementary Fig. 5). Histological assessments also indicates that neither S-RBD nor GsMTx4 impacts heart, kidney, spleen and liver of hACE2 Tg mice (Supplementary Fig. 6).

### S-RBD induces significant apoptosis and elevated [Ca^2+^]_i_, which can be inhibited by GsMTx4, in freshly isolated and cultured pulmonary microvascular endothelial cells (PMVECs) from hACE2 Tg mice

**Fig. 5:**
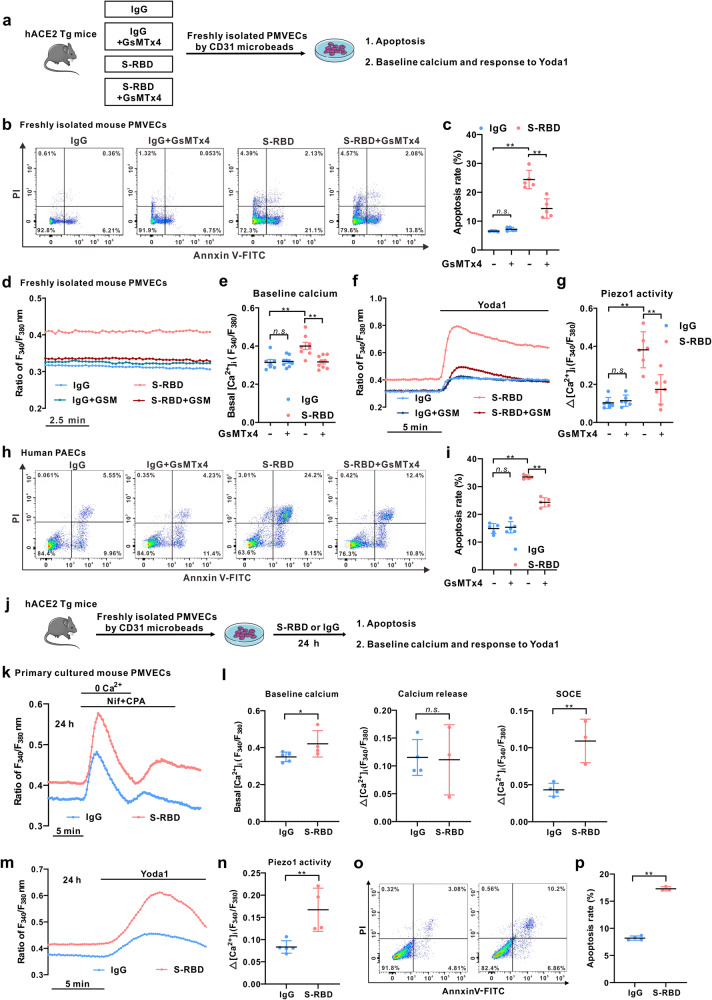
Effects of S-RBD and GsMTx4 on the intracellular calcium and apoptosis in freshly isolated and cultured pulmonary microvascular endothelial cells (PMVECs) from hACE2 Tg mice. **a** Schematic representation of the fresh isolation and experimental procedures of PMVECs from each group of mice. **b**, **c** Flow cytometry (**b**) and summarized graph (**c**) showing the apoptosis level of PMVECs freshly isolated from each group of hACE2 Tg mice. Bar values are mean ± SD, *n* = 5 experiments in each group. **P < 0.01 as indicated. **d**–**g** Representative traces and summarized data showing the effects of S-RBD and/or GsMTx4 on baseline calcium (**d**, **e**) and Yoda1-induced calcium increase (**f**, **g**) in freshly isolated PMVECs from each group of mice. Bar values are mean ± SD, *n* = 6–9 experiments in 156 (IgG), 348 (IgG + GSM), 241 (S-RBD) and 369 (S-RBD + GSM) cells. *P < 0.05, **P < 0.01 as indicated. **h**, **i** Representative flow cytometry (**h**) and summarized graph (**i**) showing the effects of S-RBD and/or GSMTx4 for 24-hour on cell apoptosis in human PAECs. Bar values are mean ± SD, *n* = 5 experiments in each group, **P < 0.01 as indicated. **j** Schematic representation of the experimental procedures in primary cultured PMVECs from each group of mice. **k**–**n** Representative traces (**k**, **m**) and summarized data (**l**, **n**) showing the effects of S-RBD and/or GsMTx4 on baseline calcium, CPA-evoked intracellular calcium release and SOCE (**k**, **l**), and Yoda1-induced calcium increase (**m**, **n**) in primary cultured PMVECs from hACE2 Tg mice. Bar values are mean ± SD, *n* = 3–4 experiments in 142 (IgG) and 111 (S-RBD) cells for SOCE and *n* = 4–5 experiments in 178 (IgG) and 141 (S-RBD) cells for baseline calcium and Yoda1 responses. *P < 0.05, **P < 0.01 as indicated. **o**, **p** Flow cytometry (**o**) and summarized graph (**p**) showing the apoptosis level of primary PMVECs from hACE2 Tg mice and treated with IgG or S-RBD. Bar values are mean ± SD, *n* = 3–4 experiments in each group. **P < 0.01 as indicated

We next determined the cell apoptosis and intracellular calcium levels using freshly isolated PMVECs from hACE2 Tg mice treated with S-RBD and/or GsMTx4 (Fig. 5a), following the culture and validation protocols shown in Supplementary Fig. 7. Freshly isolated PMVECs from S-RBD-treated mice show a significantly elevated apoptotic rate, which is normalized in PMVECs from S-RBD+GsMTx4 treatment mice (Fig. 5b, c). The baseline calcium level (Fig. 5d, e) and Piezo1-mediated calcium influx (Fig. 5f, g) are both enhanced in PMVECs from S-RBD-treated mice, which is prevented by GsMTx4 co-treatment via antagonism of Piezo1 activity. Consistent with the results obtained from freshly isolated mouse PMVECs, treatment with GsMTx4 significantly inhibits S-RBD-induced apoptosis of cultured human PAECs (Fig. 5h, i). PMVECs were isolated and cultured from normal hACE2 Tg mice and were treated with S-RBD and/or GsMTx4 (Fig. 5j). The results indicate that 24-hour exposure to S-RBD significantly enhanced baseline calcium levels (Fig. 5k, l), channel activity of SOCC (Fig. 5k, l) and Piezo1 (Fig. 5m, n), as well as cell apoptosis (Fig. 5o, p), validating the major findings observed in cultured human PAECs and freshly isolated PMVECs from S-RBD and/or GsMTx4-treated hACE2 Tg mice.

### Comparative effects of different S-RBD variants on intracellular calcium homeostasis in human PAECs

**Fig. 6:**
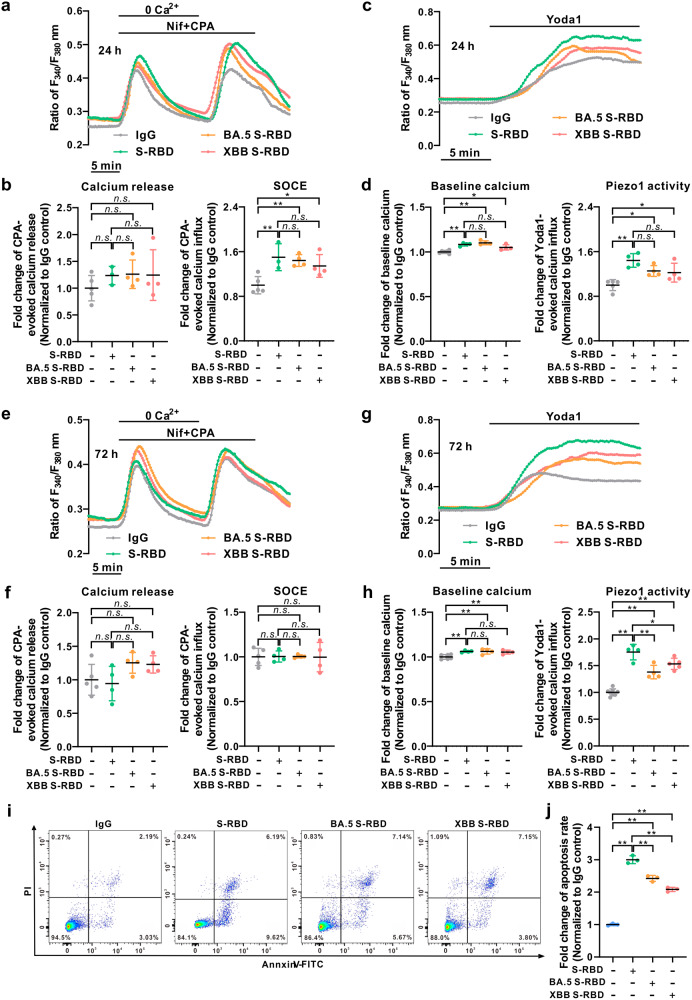
Comparative effects of Omicron S-RBD variants (BA.5.2 and XBB) on the intracellular calcium and apoptosis in human PAECs. **a**–**h** Representative traces and summarized data showing the effects of prototypic strain S-RBD and two Omicron variants of S-RBD (BA.5.2 and XBB, 4 μg/mL) for 24-hour (**a**–**d**) and 72-hour (**e**–**h**) on intracellular calcium release induced by CPA and CPA-evoked SOCE (**a**, **b**, **e**, **f**), as well as baseline calcium and Yoda1-induced calcium increase (**c**, **d**, **g**, **h**) in human PAECs. Bar values are mean ± SD, *n* = 3–5 experiments in 232 (IgG 24 h), 195 (IgG 72 h), 133 (S-RBD 24 h), 113 (S-RBD 72 h), 164 (BA.5.2 S-RBD 24 h), 113 (BA.5.2 S-RBD 72 h), 158 (XBB S-RBD 24 h), 117 (XBB S-RBD 72 h) cells for CPA-induced calcium release and SOCE measurements, *n* = 4–6 experiments in 171 (IgG 24 h), 282 (IgG 72 h), 160 (S-RBD 24 h), 120 (S-RBD 72 h), 136 (BA.5.2 S-RBD 24 h), 126 (BA.5.2 S-RBD 72 h), 118 (XBB S-RBD 24 h), 134 (XBB S-RBD 72 h) cells for Yoda1-induced calcium increase and baseline calcium measurements. ^*^P < 0.05 as indicated. **i**, **j** Representative flow cytometry (**i**) and summarized graph (**j**) showing the effects of prototypic strain S-RBD and two Omicron variants of S-RBD (BA.5.2 and XBB, 4 μg/mL) for 24-hour on cell apoptosis in human PAECs. Bar values are mean ± SD, *n* = 3 experiments in each group, **P < 0.01 as indicated

Next, we explored and compared the potential effects of different S-RBD variants, including two currently dominant Omicron variants (BA.5.2 and XBB) and two previously reported variants (Delta and Lambda), in comparison with the original prototypic strain, on intracellular calcium homeostasis in human PAECs. First, as seen in Fig. 6, prolonged exposure to Omicron BA.5.2 and XBB S-RBD for 24-hour (Fig. 6a–d) and 72-hour (Fig. 6e–h) significantly increase Yoda1-induced calcium influx through Piezo1, baseline calcium levels (Fig. 6c, d, g, h), and CPA-evoked SOCE, but not CPA-induced calcium release (Fig. 6a, b, e, f), compared to their respective IgG controls. Prolonged exposure (24-hour) to Omicron BA.5.2 and XBB S-RBD also induces significant apoptosis in cultured human PAECs (Fig. 6i, j). Notably, in comparison to the prototypic strain, Omicron variants of S-RBD (BA.5.2 and XBB) induces significantly lower levels of apoptosis (Fig. 6i, j) but similar trends in baseline calcium levels and slightly lower levels of SOCE and Piezo1 activity (Fig. 6a–h). Similar to the Omicron variants, prolonged exposure (24- and 72-hour) to Delta S-RBD (Supplementary Fig. 8a–d) or Lambda S-RBD (Supplementary Fig. 8e–h) significantly increases Piezo1 activity and baseline calcium levels, as well as the CPA-evoked SOCE but not the CPA-induced calcium release.

### Transcriptomics analysis shows the acute-to-prolonged effects of different S-RBD variants on human PAECs

We also explored the roles of three S-RBD variants on transcriptome profile using RNA-sequencing analysis in human PAECs treated with prototypic S-RBD, Delta S-RBD and Lambda S-RBD for 2-hour (acute exposure) or 24-hour (prolonged exposure). As seen in Supplementary Fig. 9–14, volcano plots, GO, and KEGG analysis show that acute exposure (2-hour) to prototypic S-RBD induces dramatic effects on PAECs, mainly affecting the biological signaling pathways related to “translation” and “mitochondria dysfunction” (Supplementary Fig. 9), which is consistent with previously reported effects.^14^ In contrast, acute exposure (2-hour) to Delta S-RBD (Supplementary Fig. 11) or Lambda S-RBD (Supplementary Fig. 13) conferred significantly less impact on PAECs, suggesting that variants of S-RBD may have evolved to substantially reduce acute damage to host cells. Prolonged exposure (24-hour) to the prototypic S-RBD (Supplementary Fig. 10), Delta S-RBD (Supplementary Fig. 12) or Lambda S-RBD (Supplementary Fig. 14) all lead to activation of signaling pathways related to “viral defense,” “immune responses” and “cell adhesion.” The differentially expressed genes in each group are shown in Supplementary Table 1.

## Discussion

This study first observed that S-RBD induces an acute-to-prolonged perturbation of intracellular calcium homeostasis in pulmonary vascular endothelial cells, mainly through acute activation of TRPV4 and prolonged upregulation of Piezo1 and Orai1, leading to the activation of these channels and persistent elevation of baseline calcium levels, promoting apoptosis of pulmonary vasculature endothelial cells. These effects can be significantly attenuated by KobA, which blocks S-RBD-ACE2 binding, and the intracellular calcium chelator BAPTA-AM. S-RBD binds to the primary receptor ACE2 and promotes its interaction with calcium channel proteins, including Piezo1, Orai1 and TRPC1, leading to the formation of ACE2-calcium channel clusters which facilitates the activation of Piezo1 and SOCC. In the hACE2 Tg mouse model, S-RBD treatment induces significant pulmonary vascular remodeling and endothelial damage, characterized by increased apoptosis and intracellular calcium regulation in PMVECs, which are attenuated by GsMTx4-induced antagonism of Piezo1 and SOCC activity. These observations provide novel mechanisms and important implications that the persistent elevation of intracellular calcium may not only cause direct damage to the pulmonary vascular endothelial cells but also provide persistent supplies of calcium to enable the viral lifecycle, driving the progressive pulmonary vascular dysfunction induced by SARS-CoV-2.

Pharmacological targeting of the intracellular calcium regulatory network may antagonize viral infection by obstructing the viral life cycle. Researchers have attempted to evaluate the potential effects of calcium channel blockers (CCBs) on COVID-19 through both experimental and retrospective analyses, although the efficacy of CCBs tends to vary across studies. Straus *et al*. found that several L-type voltage-gated calcium channel (VGCC) blockers suppress SARS-CoV-2 infection to cultured lung epithelial cells.^44^ Alsagaff et al. showed negative effects of CCBs on COVID-19 outcome^45^ but beneficial effects in hypertensive COVID-19 patients.^45,46^ Considering that current on-market CCBs mainly refer to VGCC antagonists, we believe that one possible explanation for the imperfect performance of CCBs against COVID-19 is the less likely participation of VGCC, compared to other calcium channels, during SARS-CoV-2 viral infection. Our results verified this hypothesis and showed that exposure to S-RBD induces prolonged upregulation and activation of Piezo1 and Orai1, in line with significantly increased apoptosis in human PAECs. These effects were later proven to be dependent on the S-RBD-ACE2 interaction and presence of intracellular calcium, suggesting the persistent effects of S-RBD interaction on host cells. Although studies have proposed TRPV4 in SARS-CoV-2 infection, and targeted inhibition of TRPV4 is a candidate therapeutic approach against COVID-19,^47,48^ our results indicate that acute exposure, but not prolonged exposure, to S-RBD leads to increased TRPV4 activity. Assuming that blockade of Piezo1 and SOCC could be a useful strategy to inhibit S-RBD-induced pulmonary vascular endothelial damage, we performed an in vivo animal study using the hACE2 Tg mouse model treated with S-RBD or IgG control and the Piezo1 inhibitor GsMTx4, which can also antagonize SOCC by inhibiting the channel activity of the SOCC components TRPC1 and TRPC6.^49^ These results confirm our hypothesis that inhibition of Piezo1 and SOCC by GsMTx4 efficiently prevented pulmonary vascular endothelial damage in S-RBD-treated mice. These data demonstrate an involvement of the calcium regulatory pathway in S-RBD-induced pulmonary vascular endothelial damage and provide a novel and powerful strategy against COVID-19-associated pulmonary vascular dysfunction.

As a major pathological feature of COVID-19-associated pulmonary vascular endotheliopathy, substantial microthrombi are likely induced by endothelial damage and intravascular coagulation. The vascular endothelial damage initiates inflammatory responses in the vascular microenvironment, enables the activation of innate immune cells involving platelets, monocytes and leukocytes, promotes their interaction with the endothelium, together contributing to the hypercoagulable and thrombotic state.^1^ Significantly increased plasma concentrations of von Willebrand factor (vWF) and P-selectin promote aggregation of immune cells (especially platelets) to vascular endothelium, which are in correlation with COVID-19 severity.^4^ Remarkably increased levels of key factors for vascular function and inflammation were also seen in COVID-19 patients.^50^ Similarly, results from our RNA sequencing in human PAECs also indicated that acute-to-prolonged exposure to different variants of S-RBD can drive the molecular basis that favors endothelial dysfunction, inflammation, and microthrombus aggregation in pulmonary vessels.

Many variants of SARS-CoV-2 become predominant strains including currently dominating Omicron variants.^51,52^ As is reported, Omicron variants have much higher transmission patterns and immune escape abilities than the prototypic strain and previously reported major variants.^51,53–59^ To determine whether different variants can induce different levels of intracellular calcium homeostasis and endothelial cell damage, we measured the effects of S-RBD from the prototypic strain, Omicron (BA.4/5/5.2 and BA.2/XBB), Delta, and Lambda, on intracellular calcium and regulation in cultured human PAECs. We found that prolonged exposure to various S-RBD variants, including Omicron (BA.5.2 and XBB), Delta, and Lambda, induces similar effects on baseline calcium levels and comparable effects on extracellular calcium influx through Piezo1 and SOCC, in comparison with the prototypic strain. In contrast, Omicron variants (BA.5.2 and XBB) of S-RBD exert significantly lower effects on endothelial cell apoptosis. RNA sequencing data also show obvious greater acute impacts of the prototypic strain S-RBD compared to that of the Delta and Lambda S-RBD. These results indicate the S-RBD variants may evolve to retain the ability to regulate intracellular calcium levels to benefit its viral lifecycle, while substantially reducing damages to host cells, which may explain, to some extent, the high transmissibility of Omicron variants (BA.5.2 and XBB), recently emerged as the predominant strains with a record-breaking transmissibility, enriching our understanding of Omicron. This data also sheds light on the prediction of potential long COVID complications in subjects recovered from the latest wave of Omicron infection.

Considering the continuous mutation of the virus and the evolution of advanced immune and vaccine escape abilities, the development of up-to-date vaccines is a huge project that imposes a long-lasting and tremendous burden worldwide. Therefore, the strengths of this study are the uncovering of a novel key mechanism from the host cell side that is retained in four tested major S-RBD mutants, including the currently dominant Omicron variants, and the demonstration that targeted inhibition of the S-RBD-ACE2-Piezo1/SOCC-[Ca^2+^]_i_ axis, such as blockade of Piezo1 and SOCC-mediated calcium entry by GsMTx4, are promising strategies for treating S-RBD-induced pulmonary vascular damage. Nevertheless, there are also limitations including the uncertain molecular mechanisms, such as how S-RBD activates TRPV4 and which transcriptional factors account for the progressive upregulation of Piezo1 and Orai1, even though S-RBD-induced prolonged upregulation and activation of Piezo1 and Orai1 are dependent on the S-RBD-ACE2 interaction and the presence of intracellular calcium. Future studies are desired to elucidate how S-RBD impairs pulmonary vascular endothelial cells by hijacking intracellular calcium regulatory networks. Moreover, the contribution of elevated intracellular calcium to the machinery facilitating the whole SARS-CoV-2 viral lifecycle requires verification using complete virus infection in pulmonary vascular cells and animal models.

**Fig. 7:**
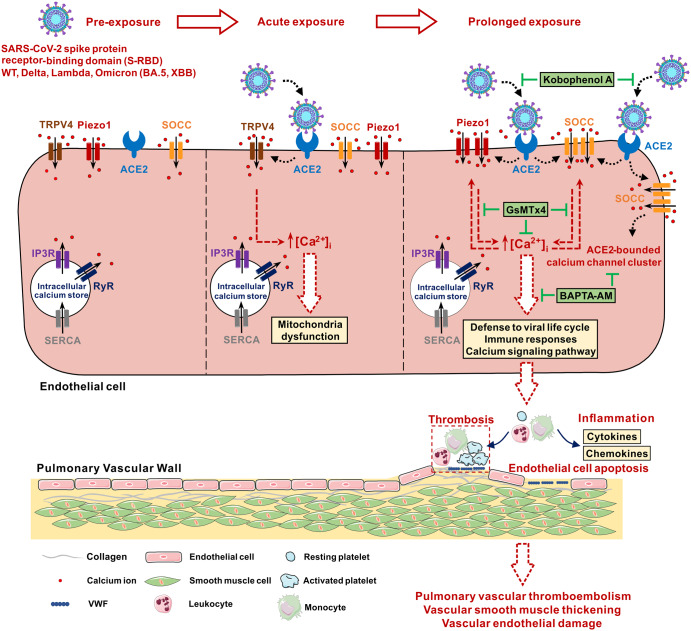
Proposed working model of the study. Illustration showing the acute-to-prolonged perturbation of S-RBD on the intracellular calcium homeostasis in endothelial cells and impairment on pulmonary vasculature. The figure was generated by CorelDRAW X8 including online picture resources from SMART - Servier Medical ART (https://smart.servier.com)

As summarized in Fig. 7, this study provides compelling pre-clinical evidence and first reports that single exposure to S-RBD is sufficient to cause acute-to-prolonged damage to the pulmonary vascular endothelium by triggering increased intracellular calcium concentrations through upregulation and activation of Piezo1 and SOCC channels. Pharmacological inhibition of these channels effectively prevented disruption of intracellular calcium homeostasis in endothelial cells, significantly eliminating structural and functional damage to the pulmonary vascular endothelium in S-RBD-treated hACE2 Tg mice. Our study strongly indicates that targeted inhibition of the ACE2-Piezo1/SOCC-[Ca^2+^]_i_ signaling axis could be a powerful strategy in treating with the pulmonary vascular damage induced by S-RBD, providing novel insights into the therapy of long COVID complications.

## Materials and methods

Full source information for chemicals and reagents, and experimental procedures are detailed in Supplementary Material.

### Animal ethic and protocols

The operation and handling protocols for the animal study were approved and reviewed by the Animal Care and Use Committee of The First Affiliated Hospital of Guangzhou Medical University (Reference number: 2020-86) and follows ARRIVE guidelines. The hACE2 Tg mice were purchased from Shanghai Model Organisms (Shanghai, China). The hACE2 Tg mice (6-week, male) were randomly grouped as: IgG + vehicle, IgG + GsMTx4, S-RBD + vehicle, S-RBD + GsMTx4. Mice were intratracheal instillation of recombinant S-RBD (5 μg/mouse/d) or IgG, together with GsMTx4 (intraperitoneal injection, 1 mg/kg/d) or vehicle (Saline) for 7 consecutive days. Inhalation of 1.5% isoflurane was used for animal anesthesia via a facemask for transthoracic echocardiography.

### Fresh isolation and culture of PMVECs from hACE2 Tg mice

Mice lungs were minced, digested with 2 mg/mL of type 2 collagenase, and gently agitated for 40 min at 37 °C. The non-ECs were excluded by CD45 microbeads (130-052-301), and then ECs were enriched by CD31 microbeads (130-097-418 for mouse cells) via magnetic separation. Isolation, culture and purity assessment protocols for mouse PMVECs are outlined in Supplementary Fig. 7. The microbeads for cell isolation were purchased from Miltenyi Biotec.

### Statistical analysis

All the values were presented in mean ± SD. The statistics were performed by Graphpad Prism 7 and SPSS 23.0. Analyses for comparison between two groups were conducted by *t*-tests; while analyses for comparison among multiple groups were conducted by one-way ANOVA and then followed by Bonferroni’s multiple comparison test or Fishers LSD post hoc test. Before analyses, normal distribution for the values were assessed by Bartlett’s test and Kolmgorov-Smirnov test. A *P* < 0.05 indicates statistically significance.

## Supplementary information

Supplementary Materials

Supplementary Table 1

## Data Availability

The raw data of RNA sequencing shown in this manuscript has been uploaded and released in the Genome Sequence Archive in National Genomics Data Center, China National Center for Bioinformation/Beijing Institute of Genomics, Chinese Academy of Sciences (GSA-Human: HRA004698, BioProject accession: PRJCA017274) that are publicly accessible at. Data supporting results of present work are available in this manuscript and supplementary materials, and also from the corresponding authors upon reasonable request.
