# Supplementary material for: SARS-CoV-2 spike protein receptor-binding domain perturbates intracellular calcium homeostasis and impairs pulmonary vascular endothelial cells
Source: Signal Transduct Target Ther. 2023 Jul 14;8:276. doi: 10.1038/s41392-023-01556-8 (PMC10349149; doi:10.1038/s41392-023-01556-8)
Supplement: Supplementary file 1 — Supplementary Materials [file 41392_2023_1556_MOESM1_ESM.doc]

Supplementary Materials for

SARS-CoV-2 Spike Protein Receptor-Binding Domain Perturbates Intracellular Calcium Homeostasis and Impairs Pulmonary Vascular Endothelial Cells

Kai Yang^1,#,*^, Shiyun Liu^1,#^, Han Yan^1,2,#^, Wenju Lu^1,#^, Xiaoqian Shan^1,3^,

Haixia Chen^1,4^, Changlei Bao^1^, Huazhuo Feng^1^, Jing Liao^5^, Shuxin Liang^1^, Lei Xu^3^,

Haiyang Tang^1^, Jason X.-J. Yuan^6^, Nanshan Zhong^1,2,*^, Jian Wang^1,2,6,*^

Correspondence to: Jian Wang (jiw037@health.ucsd.edu), Nanshan Zhong (nanshan@vip.163.com) and Kai Yang (kyang28@pku.edu.cn)

**This PDF file includes:**

Materials and Methods

Figures S1 to S14

**Materials and Methods**

**Chemicals and reagents**

Human pulmonary arterial endothelial cells (PAECs) were purchased from Lonza (Walkersville, MD, USA), cultured in a humidified atmosphere at 37°C and 5% CO_2_ with EGM-2 Endothelial Cell Growth Medium-2 containing 5% fetal bovine serum, 100 U/mL penicillin, 100 μg/mL streptomycin and EGMTM-2 Endothelial SingleQuotsTM Kit (Lonza, Walkersville, MD, USA). Cultured human PAECs of passages 4-7 were used for the cell experiments. The major reagents used in this study include: SARS-CoV-2 Spike RBD-Fc Recombinant Protein (40592-V02H, Sino Biological, Beijing, China); SARS-CoV-2 Spike RBD Omicron (BA.4/BA.5/BA.5.2) Protein (40592-V49H9-B, Sino Biological, Beijing, China); SARS-CoV-2 Spike RBD Omicron (XBB) Protein (40592-V08H144, Sino Biological, Beijing, China); SARS-CoV-2 Spike RBD Lambda mutant (L452Q, F490S) Protein (40592-V08H113, Sino Biological, Beijing, China), SARS-CoV-2 Spike RBD Delta mutant (K417N, L452R, T478K) Protein (40592-V08H115, Sino Biological, Beijing, China), SARS-CoV-2 Spike S1-His Recombinant Protein (40591-V08H, Sino Biological, Beijing, China), SARS-CoV-2 Spike S1+S2 ECD (R683A, R685A, F817P, A892P, A899P, A942P, K986P, V987P)-His Recombinant Protein (40589-V08H4, Sino Biological, Beijing, China), normal human IgG (A7001, Beyotime, Nantong, Jiangsu, China), Fc-IgG (10702-HNAH, Sino Biological, Beijing, China); Fura-2/AM, cell permeant (F1221, Thermo Fisher Scientific, Rockford, IL, USA), GsMTx4 (P1205, Selleck Chemicals LLC, Houston, TX, USA), and primary antibodies against PCNA (ab29, Abcam, Cambridge, MA, USA), TRPC1 (ab192031, Abcam, Cambridge, MA, USA); CD31 (AF3628, R&D Systems, Minneapolis, MN, USA); Cleaved-caspase 3 (#9664, Cell Signaling Technology, Danvers, MA, USA), Bax (#2772S, Cell Signaling Technology, Danvers, MA, USA), Orai1 (sc-377281, Santa Cruz Biotechnology, Santa Cruz, CA, USA), α-SMA (ab124964 and ab7817, Abcam, Cambridge, MA, USA), TRPV4 (ACC-124, Alomone labs), STIM1 (610954, BD Biosciences, San Jose, CA, USA), Bcl2 (13-8800, Thermo Fisher Scientific, Rockford, IL, USA), Piezo1 (15939-1-AP, Proteintech, Rosemont, IL, USA), ACE2 (SN0754, NOVUS, Shanghai, China), β-actin (66009-1-Ig, Proteintech, Rosemont, IL, USA) for immunoblotting or immunofluorescence staining experiments.

**Animal ethic and protocol**

All the experimental animal procedures were approved by The Animal Care and Use Committee of The First Affiliated Hospital of Guangzhou Medical University (ethical committee approval number: 2020-86) and conformed to the ARRIVE guidelines. The hACE2 Tg mice were purchased from the Shanghai Model Organisms (Shanghai, China) and raised in the Specific Pathogen Free (SPF) grade animal facility of The First Affiliated Hospital of Guangzhou Medical University. Mice were housed in a temperature-controlled facility (23-25°C) and humidity 40-60% with free access to food and water and a 12-hour light to dark cycle. The hACE2 Tg mice (6-week, male) were divided into 4 groups, including IgG + vehicle, IgG + GsMTx4, S-RBD + vehicle, S-RBD + GsMTx4. The model was established by intratracheal instillation of recombinant SARS-CoV-2 S-RBD (5 μg/mouse/d) or IgG, together with intraperitoneal injection of GsMTx4 (1 mg/kg/d) or vehicle (Saline) for 7 consecutive days. The mice were anesthetized by continuous inhalation of isoflurane (1.5%) via a facemask for transthoracic echocardiography and then overdose anesthetized and euthanized by exsanguinating after removal of the heart and lungs for subsequent histological analysis. All procedures were performed to ensure the animals’ welfare and minimize the pain during the animal protocols. The experimental operation and analysis were performed in a blinded way.

**Hemodynamic assessment**

The right ventricular systolic pressure (RVSP) was assessed by right heart catheterization (RHC) according to previously described methods. In specific, mice were anesthetized by continuous inhalation of isoflurane (1.5%) and RHC was conducted to measure right ventricle pressure (RVP) using a mouse pressure volume (PV) catheter (Millar Instruments, PVR1030, Houston, TX, USA) inserted into the RV via the external right jugular vein. Baseline calibration was performed for the catheter before each measurement to ensure that the basal pressure was zero. RVP was recorded and analyzed using the AD Instruments Lab Chart software. The right ventricular hypertrophy was reflected by Fulton Index, calculated by the mass ratio of right ventricle to left ventricle plus septum [RV/(LV+S)].

**Transthoracic echocardiography**

The cardiac function of each group of mice were assessed by functional rodent echocardiography. Mice were anesthetized by using inhaled isoflurane (1.5%) via a facemask and then subjected to transthoracic echocardiographic analysis by using a VisualSonics Vevo 2100 system (VisualSonics Inc., Toronto, ON, Canada) and a transducer (MS-400, 20-46 MHz). Specific indexes including the pulmonary acceleration time (PAT) and pulmonary ejection time (PET), changes in RV fractional area (RV FAC), tricuspid annular plane systolic excursion (TAPSE), right ventricular end-diastolic wall thickness (RVEDWT), right ventricular end-systolic wall thickness (RVESWT), ejection fraction (EF), cardiac output (CO) and left ventricular fractional shortening (LVFS) were measured and analyzed, respectively. Echocardiographic analyses were performed offline by an investigator blinded to the treatment of the mice.

**Culture of human PAECs**

Human PAECs were purchased from Lonza (Walkersville, MD, USA), cultured in a humidified atmosphere at 37°C and 5% CO_2_ with EGM-2 Endothelial Cell Growth Medium-2 (Lonza, Walkersville, MD, USA). Cultured PAECs of passages 4-7 were used for the cell experiments that treated with different variants of S-RBD for 1-, 2- or 24-hour. For the 72-hour treatment, S-RBD was removed after 24-hour exposure by replacing culture medium and the cells were harvested after 48-hour to investigate the prolonged subsequent effects of S-RBD exposure to host cells.

**Fresh isolation of pulmonary microvascular endothelial cells (PMVECs) from hACE2 Tg mice**

Lungs collected from euthanized mice were minced, digested with 2 mg/mL of type 2 collagenase, and gently agitated for 40-min at 37℃. The cells were re-suspended, filtered through a 40 μm cell strainer and centrifuged at 600 g for 5-min at 4℃. To exclude non-ECs, cell samples were re-suspended in 500 μL of 0.1% bovine serum albumin (BSA) and incubated with 10 μL of CD45 microbeads (130-052-301, Miltenyi Biotec, Bergisch Gladbach, Germany). After incubation for 15-min on ice, cell suspension was added onto the LS columns (130-042-401, Miltenyi Biotec, Bergisch Gladbach, Germany) and washed with 0.1% BSA for three times. The effluents were collected and incubated with CD31 microbeads (130-097-418 for mouse cells, MiltenyiBiotec) for 15-min on ice. LS columns were used to collect the cells that were positively labeled by CD31 microbeads via magnetic separation according to the manufacturer’s instructions. For fresh isolation experiments, PMVECs were directly subjected to assessments of apoptosis, immunofluorescent staining and calcium measurements, maximally retaining the *in vivo* cell phenotype. For primary culture experiments, PMVECs isolated from normal hACE2 Tg mice were serum cultured in EGM-2 Endothelial Cell Growth Medium-2 (Lonza, Walkersville, MD, USA) and treated with S-RBD and/or GsMTx4. The purity for isolated PMVECs was validated by positive immunofluorescent staining of specific endothelial cell marker CD31 and negative staining of specific smooth muscle cell marker α-SMA. Cells with >95% purity were used for consequent experiments. The protocols for mouse PMVECs isolation, culture and purity validation were outlined in Supplementary **Fig. 7**.

**Plasmid transfection**

ACE2 overexpression plasmid vector pCMV3-Flag-ACE2 that encodes full-length human ACE2 and empty vector pCMV3-SP-N-FLAG-NCV (pCMV3-empty) were obtained from Sino Biological Inc. (Beijing, China). Cultured HEK293T grown to 60-70% confluence in 10-cm culture plate were transfected with either pCMV3-Flag-ACE2 (5 μg) or pCMV3-SP-N-FLAG-NCV (5 μg) by using Lipofectamine 3000 (Invitrogen, Thermo Fisher Scientific) that was prepared in Opti-MEM media (GIBCO, Thermo Fisher Scientific) according to the manufacturer’s instructions. After 48-hour transfection, cells were treated with or without IgG, S-RBD, Kobophenol A (HY-126419, MedChemExpress, Monmouth Junction, NJ) and BAPTA-AM (ab120503, Abcam, Cambridge, MA, USA) for 24-hour. Cells were harvested and proceeded for co-immunoprecipitation assay.

**Co-immunoprecipitation assay**

HEK293T cells were transfected with indicated plasmids with Lipofectamine 3000 (Invitrogen, Thermo Fisher Scientific). In brief, cell lysates were pre-incubated with protein A/G agarose (Santa Cruz Biotechnology) at 4℃ for 1-hour and the supernatants were collected. Then lysates were incubated with protein A/G agarose at 4℃ for 2-hour, and then incubated with anti-Flag antibody (#8146, Cell Signaling Technology, Danvers, MA, USA) or IgG antibody (Beyotime, Shanghai, China) overnight at 4℃. Subsequently, protein A/G agarose was recollected via centrifuge and washed to remove non-specifically bounded proteins. The bounded proteins were eluted with elution buffer for western blot analysis.

**Real-Time RT-PCR (RT-qPCR)**

Total RNAs were isolated by using RNeasy plus mini kit (QIAGEN, Chatsworth, CA). Reverse transcription was performed with RT2 First Strand Kit (QIAGEN) with a reaction system containing 1 µg of total RNA in a 20 µL volume. Then RT-qPCR was performed with Scofast TM EvaGreen SuperMix (Bio-Rad, Hercules, CA) in a CFX96 TM real-time system (Bio-Rad) according to protocols: Initial enzyme activation at 95°C for 3-min, followed by 40 cycles at 95°C for 5-sec and at 60°C for 15-sec. The relative concentration of each transcript was calculated with Pfaffl method[^1^](#_ENREF_1). The primer sequences used include: *hPiezo1*: Forward: 5’-TTCCCCAACAGCACCAACTT-3’, Reverse: 5’-CACGATGGCCTCGAATACCA-3’; *hTRPC1*: Forward: 5’-AGTGACGAGCCTCTTGACAA-3’, Reverse: 5’-CTTCTTACAGGTGGGCTTGC-3’; *hSTIM1*: Forward: 5’-CACACTCTTTGGCACCTTCC-3’, Reverse: 5’-CCAGCTGGGGTCTATGTTGA-3’; *hOrai1*: Forward: 5’-TTATCGTCTTCGCCGTCCAC-3’, Reverse: 5’-TCCTGTAAGCGGGCAAACTC-3’; *hTRPV4*: Forward: 5’-CTCCAAGGAGAGCAAGCACA-3’, Reverse: 5’-CCAGTTCACCTCATCCACCC-3’; *h18S*: Forward: 5’-GCAATTATTCCCCATGAACG-3’, Reverse: 5’-GGCCTCACTAAACCATCCAA-3’. The *h18S* ribosomal RNA was used as housekeeping gene throughout the experiments.

**RNA extraction, library construction and RNA sequencing**

Total RNAs were extracted from human PAECs treated with IgG or different variants of S-RBD by using TRlzol (Life technologies, CA, USA). RNA quality and concentration were determined by an Agilent 2100 Bioanalyzer (Agilent Technologies, Inc., Santa Clara, CA, USA). The mRNA was isolated by NEBNext Poly(A) mRNA Magnetic Isolation Module (E7490, NEB, Ipswich, MA, USA). The cDNA library was constructed following the manufacturer’s instructions of NEBNext Ultra RNA Library Prep Kit for Illumina (E7530, NEB, Ipswich, MA, USA) and NEBNext Multiplex Oligos for Illumina (E7500, NEB, Ipswich, MA, USA). The constructed cDNA libraries were sequenced on an Illumina HiSeq™ sequencing platform (Biomarker Technologies, China). Transcriptome analysis was performed using reference genome-based reads mapping low quality reads, such as only adaptor, unknown nucleotides > 5%, or Q20 < 20% (percentage of sequences with sequencing error rates < 1%), were removed by perl script. The clean reads that were filtered from the raw reads were mapped using Tophat2 software[^2^](#_ENREF_2). The aligned records from the aligners in BAM/SAM format were further examined to remove potential duplicate molecules. Gene expression levels were estimated using FPKM values (fragments per kilobase of exon per million fragments mapped) by the Cufflinks software[^3^](#_ENREF_3). Identification of differential gene expression[^4^](#_ENREF_4) and Q-value were used to evaluate differential gene expression between IgG and S-RBD treatment groups. After that, gene abundance differences between samples were calculated based on the ratio of the 1FPKM values. The false discovery rate (FDR) control method was used to identify the threshold of the P-value in multiple tests in order to compute the significance of the differences. Genes were retrieved based on the best BLAST hit (highest score) along with their protein functional annotation. To annotate the gene with gene ontology (GO) terms, the Nr BLAST results were imported into the Blast2 GO program[^5^](#_ENREF_5). GO annotations for the genes were obtained by Blast2GO. The gene sequences were also aligned to the Clusters of Orthologous Group (COG) database to predict and classify functions[^6^](#_ENREF_6). KEGG pathways were assigned to the assembled sequences by perl script. The raw sequence data reported in this paper have been deposited and released in the Genome Sequence Archive[^7^](#_ENREF_7) in National Genomics Data Center[^8^](#_ENREF_8), China National Center for Bioinformation/Beijing Institute of Genomics, Chinese Academy of Sciences (GSA-Human: HRA004698, BioProject accession: PRJCA017274) that are publicly accessible at https://ngdc.cncb.ac.cn/gsa-human/browse/HRA004698.

**Immunofluorescent staining**

To better visualize and quantify distal PA smooth muscle muscularization and remodeling, as well as loss of PA endothelium and smooth muscle proliferation. Lung sections were deparaffinized and dehydrated, followed by antigen retrieval by boiling in EDTA antigen retrieval buffer (pH 8.0). Sections were then incubated with permeabilization solution (Beyotime, Shanghai, China) for 10-min at room temperature. Using blocking solution (Beyotime, Shanghai, China) to incubate at 37°C for 40-min, slides were incubated with antibodies against smooth muscle specific marker α-SMA and endothelial marker CD31, together with the apoptotic marker Cleaved-caspase 3, the proliferation marker PCNA, and phosphorylated Ca^2+^/calmodulin-dependent protein kinase II (p-CaMKII) were incubated overnight at 4°C, followed by incubation with the secondary fluorescent antibody for 1-hour. Nuclei were counterstained with DAPI. Lung sections were imaged with a fluorescence microscope scanning system (Panoramic DESK, 3DHISTECH ltd. Budapest, Hungary). The positive signals of VWF (VWF+), α-SMA (α-SMA+), CD31 (CD31+), cleaved caspase 3 (Cleaved-cas 3+) and PCNA (PCNA+) were captured in distal PA of mice from each group. The mean intensity of PCNA+ signal in smooth muscle layer, and p-CaMKII+ and Cleaved-cas 3+ signals in endothelial layer were calculated by using Image-J software.

**Small interfering RNA (siRNA) transfection and knockdown**

According to the manufacture’s instruction, the cells were transiently transfected with non-targeting siRNA (GenePharma, Shanghai, China) and siRNAs against Piezo1, Orai1 and STIM1 (GenePharma, Shanghai, China) by using GeneSilencer siRNA Transfection Reagent (T500750, GeneSilencer, Abingdon, UK) for 72-hour, then measurement of intracellular calcium concentration ([Ca^2+^]_i_), western blot, contraction and proliferation assay were performed accordingly.

**Western blot**

After each treatment, cells were lysed using 1× RIPA buffer (GBCBIO Technologies, Guangzhou, China) containing protease inhibitor cocktail (Sigma-Aldrich, St. Louis, MO, USA) and PMSF (GBCBIO Technologies, Guangzhou, China). Cells in lysis buffer were centrifuged at 12,000 rpm for 30-min at 4°C to isolate the lysate. Protein concentration was quantified using Pierce™ BCA Protein Assay Kit (Thermo Fisher Scientific, Rockford, IL, USA). Lysates with equal quantities of protein were mixed and boiled in 5× loading buffer (Boston BioProducts, Ashland, MA, USA). Protein lysates were resolved by sodium dodecyl sulfate-polyacrylamide gel electrophoresis (SDS-PAGE) and transferred onto 0.45 μm nitrocellulose membranes (Bio-Rad, Hercules, CA, USA). Membranes were incubated for 2-hour at room temperature in a blocking buffer [0.1% Tween 20 in TBS (TBST)] containing 5% nonfat dry milk powder. The membranes were then incubated with primary antibodies in 3 mL of primary antibody dilution buffer (Beyotime, Shanghai, China) with shaking overnight at 4°C. Membranes were washed three times with TBST for 10-min each, followed by incubation in secondary antibody conjugated to horseradish peroxidase for 1-hour at room temperature in secondary antibody dilution buffer. Membranes were washed three times for 10-min each, and blot image were captured by Tanon 5200 automatic digital gel image analysis system (Tanon, Shanghai, China). Intensities for the target protein blots were normalized to β-tubulin, serving as internal controls. Band intensity was quantified by ImageJ.

**Cell apoptosis assay**

The cell apoptosis of human PAECs and mice PMVECs was measured by flow cytometry using TransDetect Annexin V-FITC/PI Cell Apoptosis Detection Kit (TransGen Biotech, Beijing, China) according to the manufacturer’s instructions. Briefly, cells were harvested and washed once in cold phosphate‐buffered saline and then stained with Annexin-V reagent and propidium iodide (PI) for 30-min in dark. After staining, the cells were analyzed by flow cytometry (BD Biosciences, San Jose, CA, USA).

**Intracellular calcium imaging measurement**

Cells were grown at 60%-70% confluence on 25-mm diameter circular glass coverslips and were incubated with 2.8 μg/mL Fura-2/AM in Krebs Ringer bicarbonate (KRB) buffered solution for 40-min in 37°C incubator. Cells were perfused at 1 mL/min for 10-min to remove the extracellular dye, and [Ca^2+^]_i_ measurement was determined at 12-sec intervals from the ratio of F340/F380 at room temperature. The percentage decline in Fura-2 density reflects the activity of channels at plasma membrane. KRB buffered extracellular solution contained (in mM) 120 NaCl, 4.3 KCl, 1.8 CaCl_2_, 1.2 MgCl_2_, 10 Glucose, 19 NaHCO_3_, 1.1 KH_2_PO_4_ (pH was adjusted to 7.35-7.45 with 4 M NaOH). The Ca^2+^-free solution was prepared by replacing 1.8 mM CaCl_2_ with equimolar MgCl_2_ and adding 0.1 mM EDTA to chelate residual Ca^2+^. Yoda1 (Sigma-Aldrich, St. Louis, MO, USA), Cyclopiazonic acid (Sigma-Aldrich, St. Louis, MO, USA), GSK2193874 (Selleck Chemicals LLC, Houston, TX, USA) were prepared and aliquoted as stock solutions and stored at -20°C until use.

**Statistical analysis**

Graphpad Prism 7 and SPSS (version 23.0) were used to perform statistical analyses. Data were tested for normal distribution (Kolmgorov-Smirnov test) and/or homogeneity of variance (Bartlett’s test). Differences between 2 groups were assessed with the t-test; while for ≥3 groups, differences were assessed by the one-way ANOVA followed by Bonferroni’s multiple comparison test or Fishers LSD post hoc test. The data were represented as mean ± SD and sample size (n) refers to the number of animals or independent cell culture experiments, which was described in detail in each figure legend. A value of *P* < 0.05 is considered statistically significant.

**Reference**

1. Pfaffl MW. A new mathematical model for relative quantification in real-time rt-pcr. *Nucleic Acids Res*. **29**:e45 (2001)

2. Kim D, et al. Tophat2: Accurate alignment of transcriptomes in the presence of insertions, deletions and gene fusions. *Genome Biol*. **14**:R36 (2013)

3. Trapnell C, et al. Transcript assembly and quantification by rna-seq reveals unannotated transcripts and isoform switching during cell differentiation. *Nat Biotechnol*. **28**:511-515 (2010)

4. Anders S, et al. Differential expression analysis for sequence count data. *Genome Biol*. **11**:R106 (2010)

5. Conesa A, et al. Blast2go: A universal tool for annotation, visualization and analysis in functional genomics research. *Bioinformatics*. **21**:3674-3676 (2005)

6. Tatusov RL, et al. The cog database: A tool for genome-scale analysis of protein functions and evolution. *Nucleic Acids Res*. **28**:33-36 (2000)

7. Chen T, et al. The genome sequence archive family: Toward explosive data growth and diverse data types. *Genomics Proteomics Bioinformatics*. **19**:578-583 (2021)

8. Database resources of the national genomics data center, china national center for bioinformation in 2022. *Nucleic Acids Res*. **50**:D27-D38 (2022)

**Supplementary Figures**

**
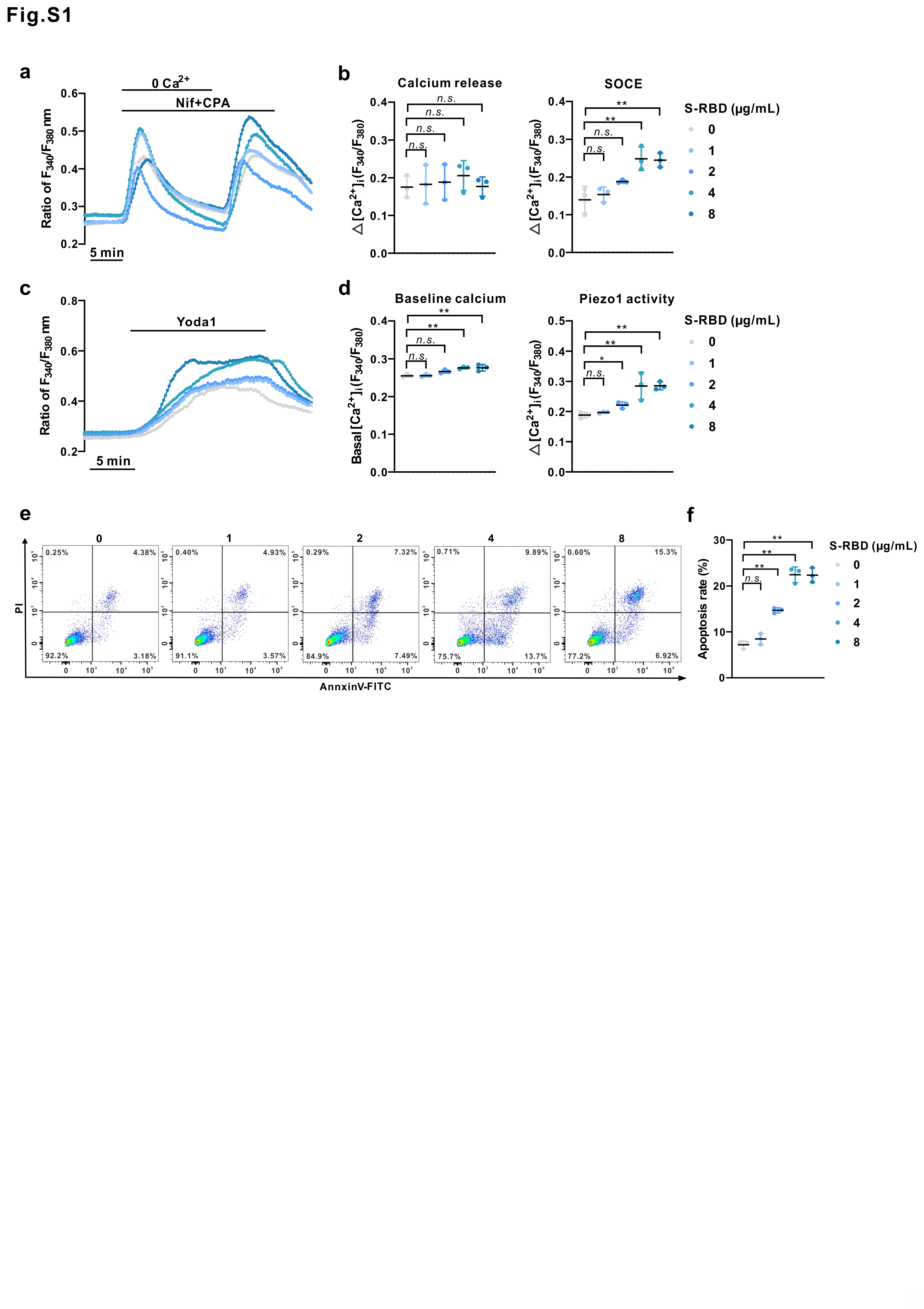
**

**Supplementary Figure 1:** **Dose-dependent effects of SARS-CoV-2 S-RBD on calcium responses and cell apoptosis in human PAECs.**

**a-d:** Representing traces (**a, c**) and summarized data (**b, d**) showing the effects of S-RBD (1, 2, 4 and 8 μg/mL) for 24-hour on intracellular calcium release induced by CPA (10 μM) and CPA-evoked SOCE (**a, b**), baseline calcium and Yoda1 (0.5 μM)-induced calcium increase (**c, d**) in cultured human PAECs. Bar values are mean ± SD, n = 3 experiments in each group. **^*^***P* < 0.05, **^**^***P* < 0.01 and “n.s.” indicates no significant difference. **e, f:** Representing flow cytometry (**e**) and graph (**f**) showing the effects of S-RBD (0, 1, 2, 4 and 8 μg/mL) for 24-hour on cell apoptosis in human PAECs. Bar values are mean ± SD, n = 3 experiments in each group. **^**^***P* < 0.01 and “n.s.” indicates no significant difference.

**
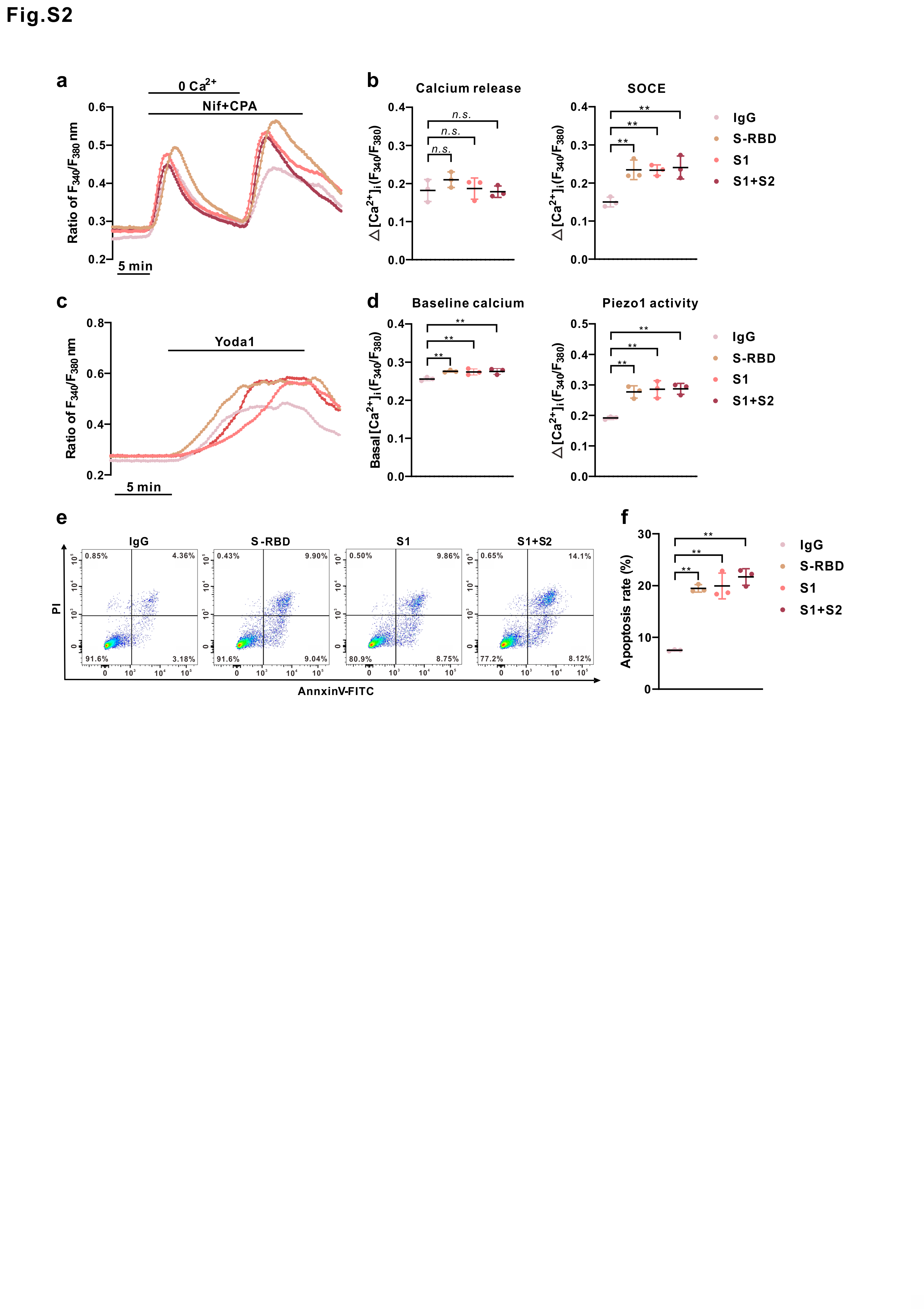
**

**Supplementary Figure 2:** **Comparative effects of S-RBD, S1 and S1+S2 on calcium responses and cell apoptosis in human PAECs.**

**a-d:** Representing traces (**a, c**) and summarized data (**b, d**) showing the effects of S-RBD, S1 and S1+S2 (4 μg/mL) for 24-hour on intracellular calcium release induced by CPA (10 μM) and CPA-evoked SOCE (**a, b**), baseline calcium and Yoda1 (0.5 μM)-induced calcium increase (**c, d**) in cultured human PAECs. Bar values are mean ± SD, n = 3 experiments in each group. **^**^***P* < 0.01 and “n.s.” indicates no significant difference. **e, f:** Representing flow cytometry (**e**) and graph (**f**) showing the effects of S-RBD, S1 and S1+S2 (4 μg/mL) for 24-hour on cell apoptosis in human PAECs. Bar values are mean ± SD, n = 3 experiments in each group. **^**^***P* < 0.01 and “n.s.” indicates no significant difference.

**
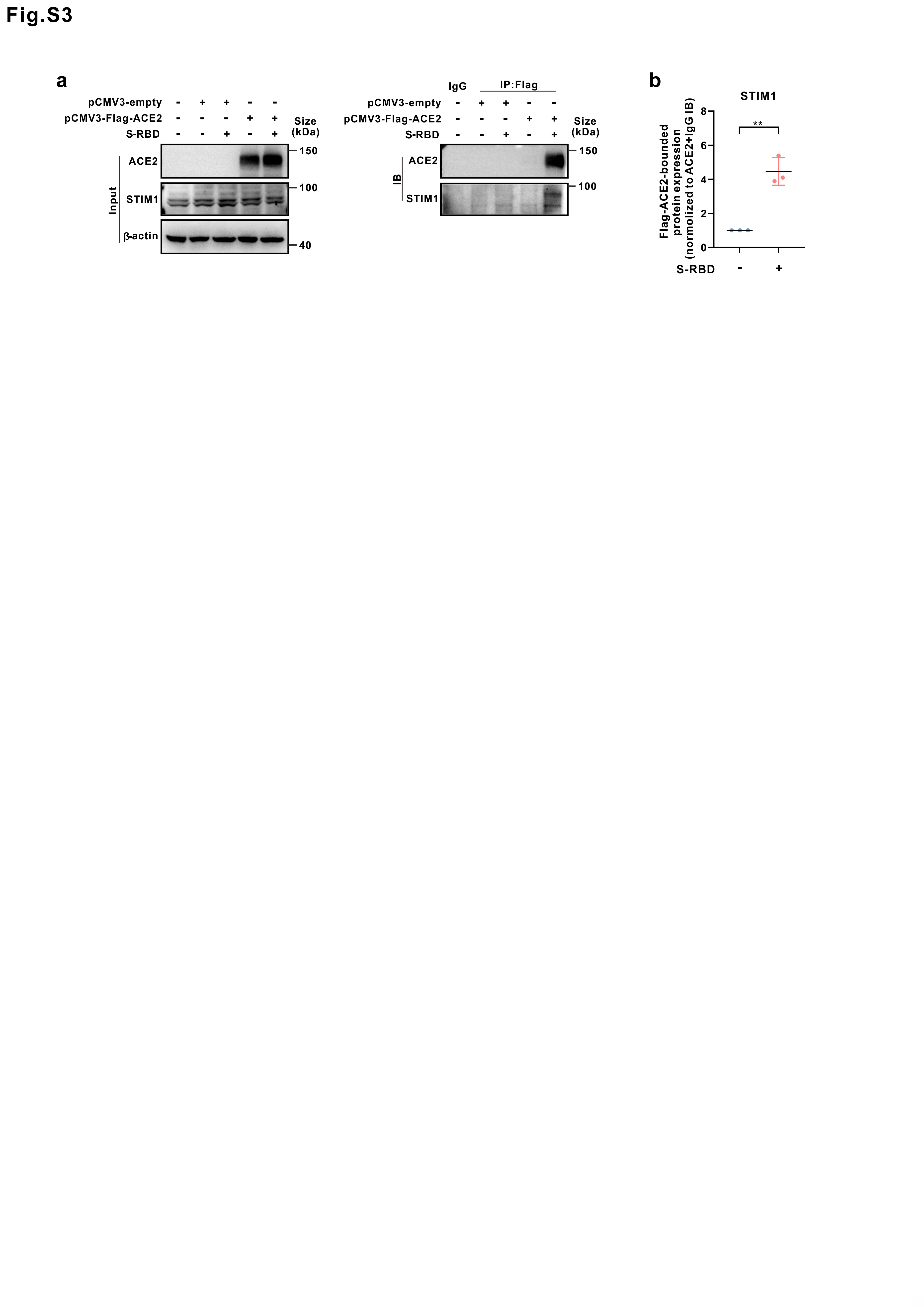
**

**Supplementary Figure 3:** **STIM1 participates in S-RBD/ACE2-induced cluster formation in human PAECs.**

**a, b:** Representing Co-IP (**a**) and analyzed bar graph (**b**) showing the expression of STIM1 in Flag-ACE2-bounded fractions from pCMV3-Flag-ACE2-transfected HEK293T treated with IgG or S-RBD for 24-hour. Bar values are mean ± SD, n = 3 experiments in each group. **^**^***P* < 0.01 as indicated.

**
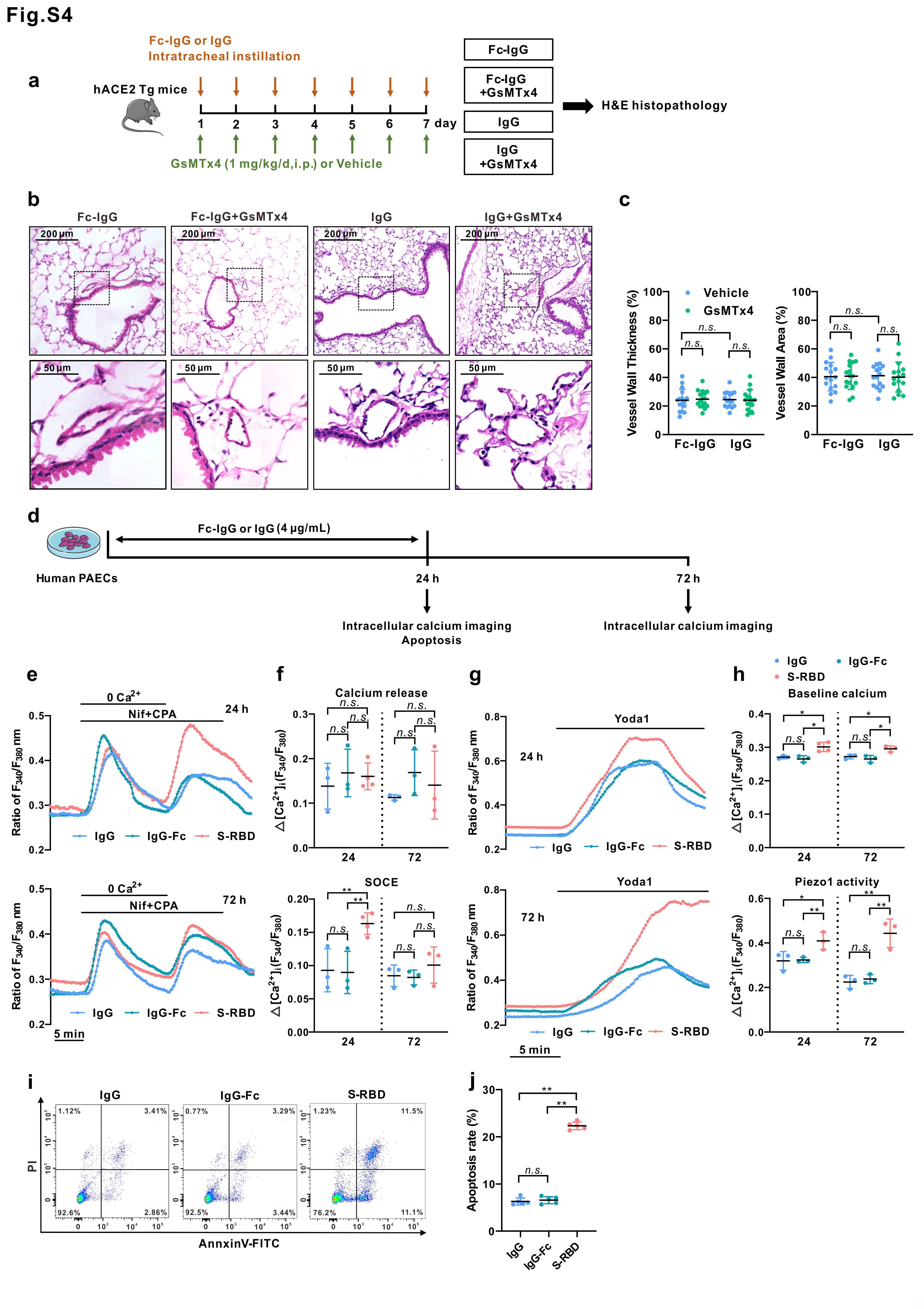
**

**Supplementary Figure 4:** **Comparative *in vivo* and *in vitro* effects of Fc-IgG and IgG.**

**a:** Schematic representation of the *in vivo* animal experimental design. **b, c:** Representing H&E staining (**b**) and analyzed bar graphs (**c**) showing the % thickness and wall area of pulmonary vasculature in hACE2 Tg mice treated with Fc-IgG/IgG with or without GsMTx4 for 7-day. Graph values are mean ± SD, n = 15-20 vessels from 5 mice in each group. The “n.s.” indicates no significant difference. **d:** Schematic representation of the *in vitro* cell experimental design. **e-h:** Representing traces (**e, g**) and summarized data (**f, h**) showing the effects of Fc-IgG, IgG and S-RBD (4 μg/mL) for 24-hour and 72-hour on intracellular calcium release induced by CPA (10 μM) and CPA-evoked SOCE, baseline calcium and Yoda1 (0.5 μM)-induced calcium increase in cultured human PAECs. Bar values are mean ± SD, n = 3-4 experiments in each group. **^*^***P* < 0.05 and “n.s.” indicates no significant difference. **i, j:** Representing flow cytometry (**i**) and graph (**j**) showing the effects of Fc-IgG, IgG and S-RBD (4 μg/mL, 24-hour) on cell apoptosis in human PAECs. Bar values are mean ± SD, n = 5 experiments in each group. **^**^***P* < 0.01 and “n.s.” indicates no significant difference.

**
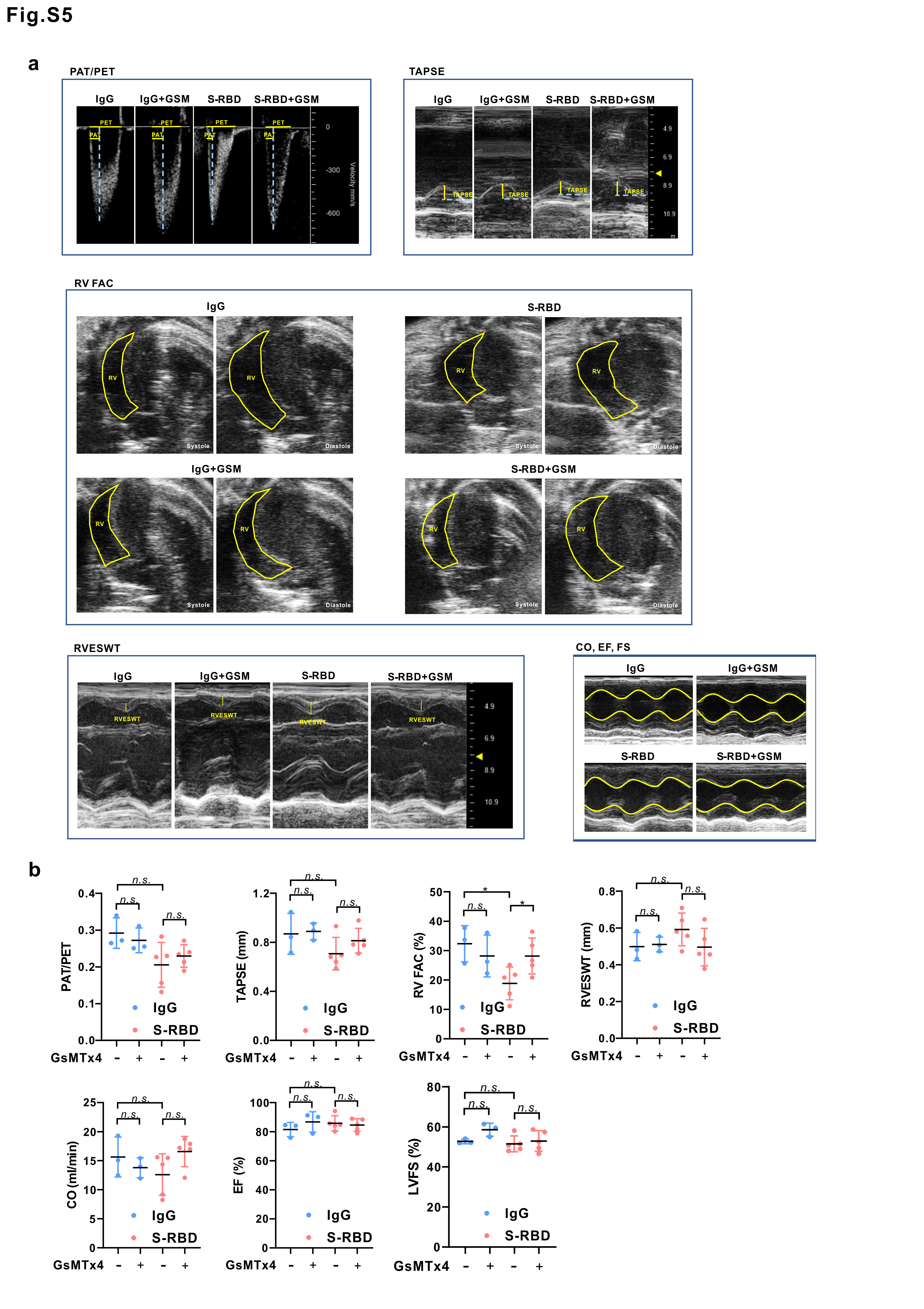
**

**Supplementary Figure 5:** **Echocardiographic analysis showing the effects of S-RBD and/or GsMTx4 on heart function in hACE2 Tg mice.**

**a, b:** Showing the typical original images (**a**) and summarized bar graphs (**b**) of the echocardiographic indexes including the pulmonary acceleration time/pulmonary ejection time (PAT/PET), right ventricle fractional area change (RV FAC), tricuspid annular plane systolic excursion (TAPSE), right ventricular end-systolic wall thickness (RVESWT), right ventricular end-diastolic wall thickness (RVEDWT), cardiac output (CO), ejection fraction (EF) and left ventricular fractional shortening (LVFS) in hACE2 Tg mice treated with S-RBD and/or GsMTx4 for 7-day. Graph values are mean ± SD. **^*^***P* < 0.05 and “n.s.” indicates no significant difference, n = 3-5 in each group.

**Supplementary Figure 6:** **Histological analysis showing the potential effects of S-RBD and/or GsMTx4 treatments on major organs in hACE2 Tg mice.**

**a, b:** Showing the potential effects of SARS-CoV-2 S-RBD and/or GsMTx4 treatments on major organs including heart (**a**), liver, spleen and kidney (**b**) in hACE2 Tg mice treated with S-RBD and/or GsMTx4 for 7-day. Typical H&E staining images represent data from n = 3-5 mice in each group.

**
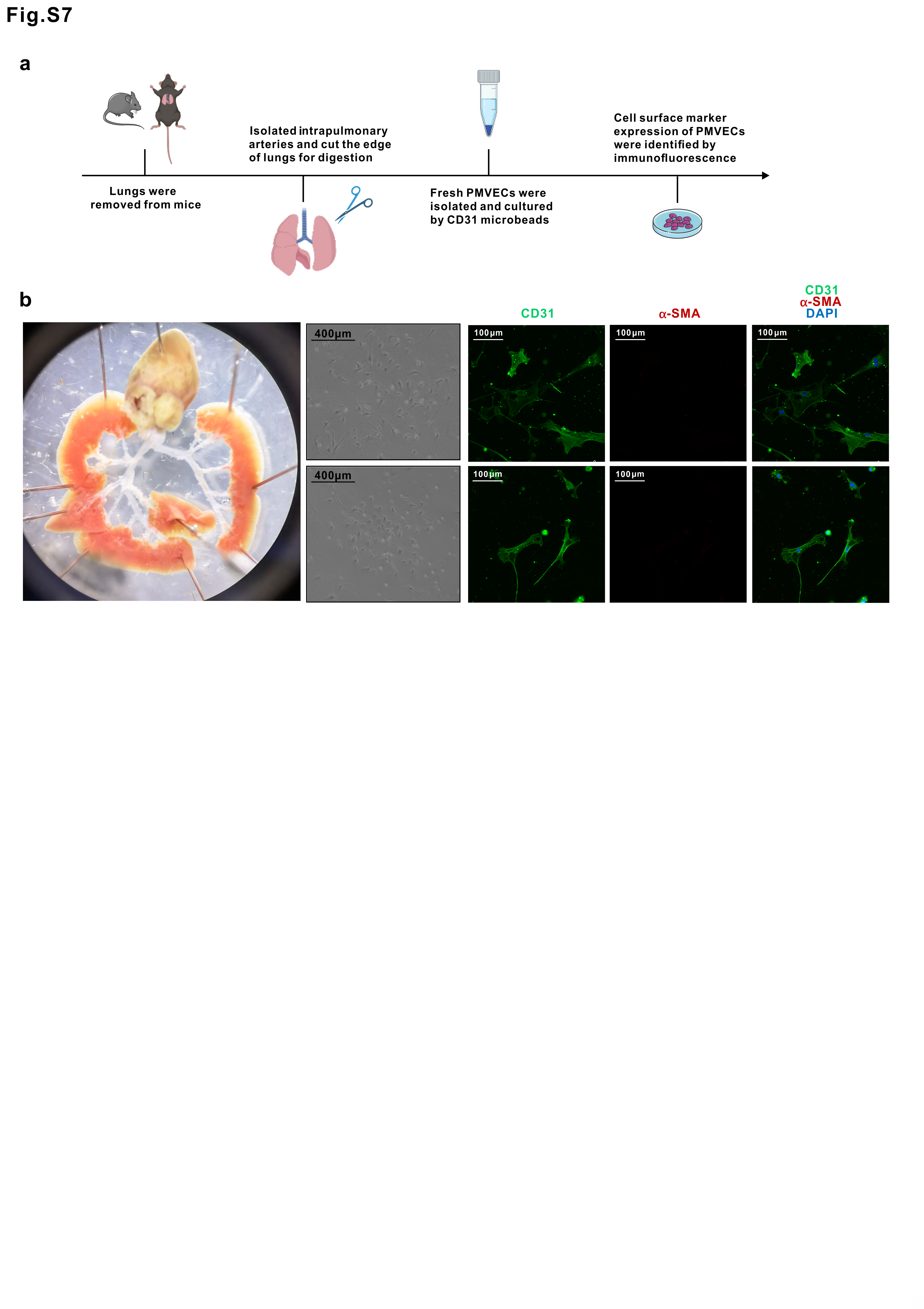
**

**Supplementary Figure 7:** **Isolation, culture and validation protocols for primary pulmonary microvascular endothelial cells (PMVECs) from hACE2 Tg mice.**

**a, b:** Schematic representation (**a**) and images (**b**) showing the isolation, culture and purity validation protocols for PMVECs from hACE2 Tg mice.

**
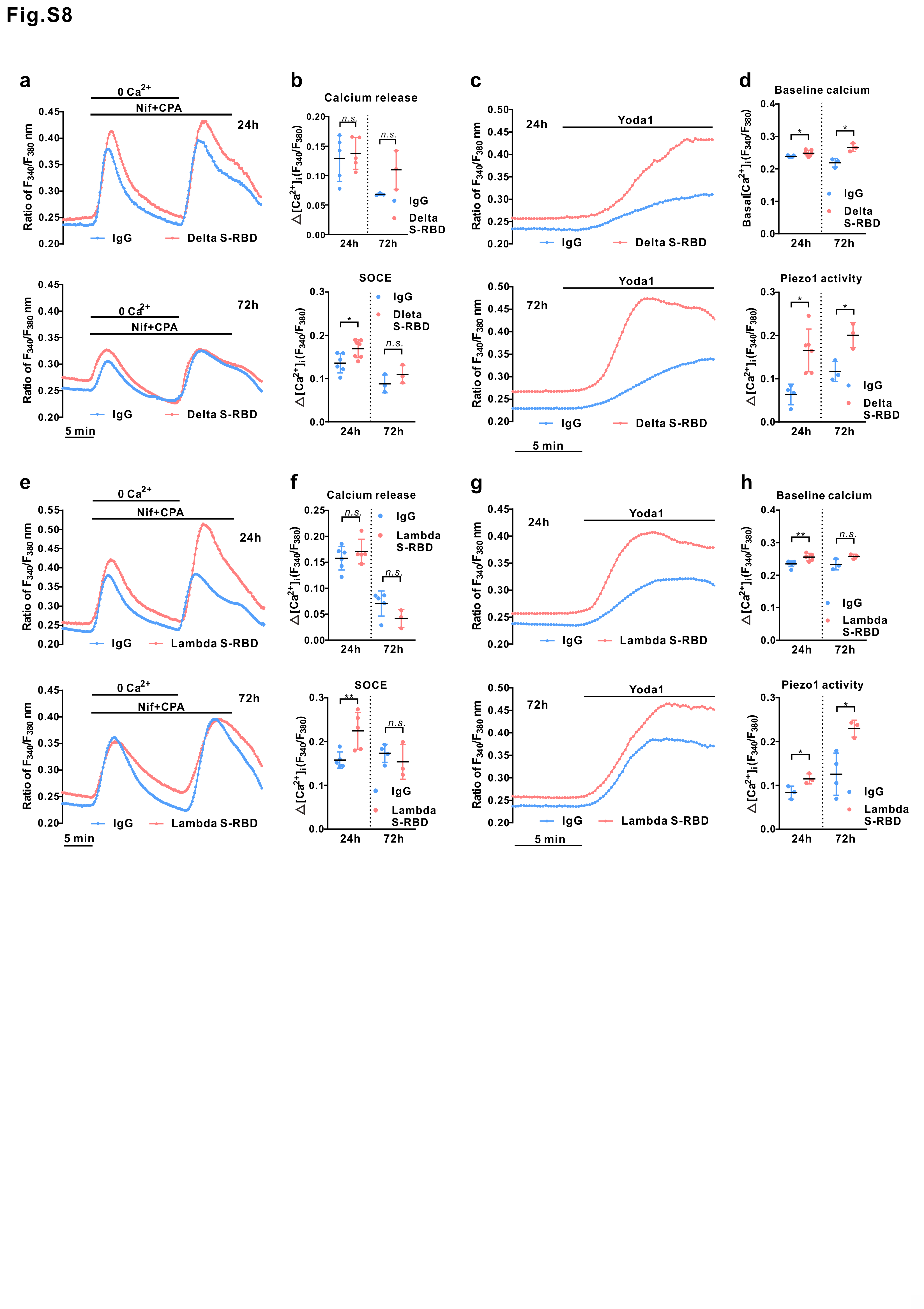
**

**Supplementary Figure 8:** **Comparative effects of different S-RBD variants (Delta and Lambda) on the intracellular calcium regulation in human PAECs.**

**a-d:** Representing traces (**a, c**) and summarized data (**b, d**) showing the effects of Delta variant of S-RBD (4 μg/mL for 24- and 72-hour, Delta S-RBD) on intracellular calcium release induced by CPA and CPA-evoked SOCE, as well as baseline calcium and Yoda1-induced calcium increase in human PAECs. Bar values are mean ± SD, n = 3-7 experiments in 226 (IgG 24h), 129 (IgG 72h), 211 (Delta S-RBD 24h) and 124 (Delta S-RBD 72h) cells for CPA-induced calcium release and SOCE measurements. Bar values are mean ± SD, n = 3-6 experiments in 214 (IgG 24h), 125 (IgG 72h), 245 (Delta S-RBD 24h) and 120 (Delta S-RBD 72h) cells for Yoda1-induced calcium increase and baseline calcium measurements. **^*^***P* < 0.05 as indicated. **e-h:** Representative traces (**e, g**) and summarized data (**f, h**) showing the effects of Lambda variant of S-RBD (4 μg/mL for 24- and 72-hour, Lambda S-RBD) on intracellular calcium release induced by CPA and CPA-evoked SOCE, as well as baseline calcium and Yoda1-induced calcium increase in human PAECs. Bar values are mean ± SD, n = 3-6 experiments in 248 (IgG 24h), 224 (IgG 72h), 123 (Lambda S-RBD 24h) and 120 (Lambda S-RBD 72h) cells for CPA-induced calcium release and SOCE measurements. Bar values are mean ± SD, n = 3-7 experiments in 288 (IgG 24h), 170 (IgG 72h), 188 (Lambda S-RBD 24h) and 122 (Lambda S-RBD 72h) cells for Yoda1-induced calcium increase and baseline calcium measurements. **^*^***P* < 0.05, **^**^***P* < 0.01 as indicated.


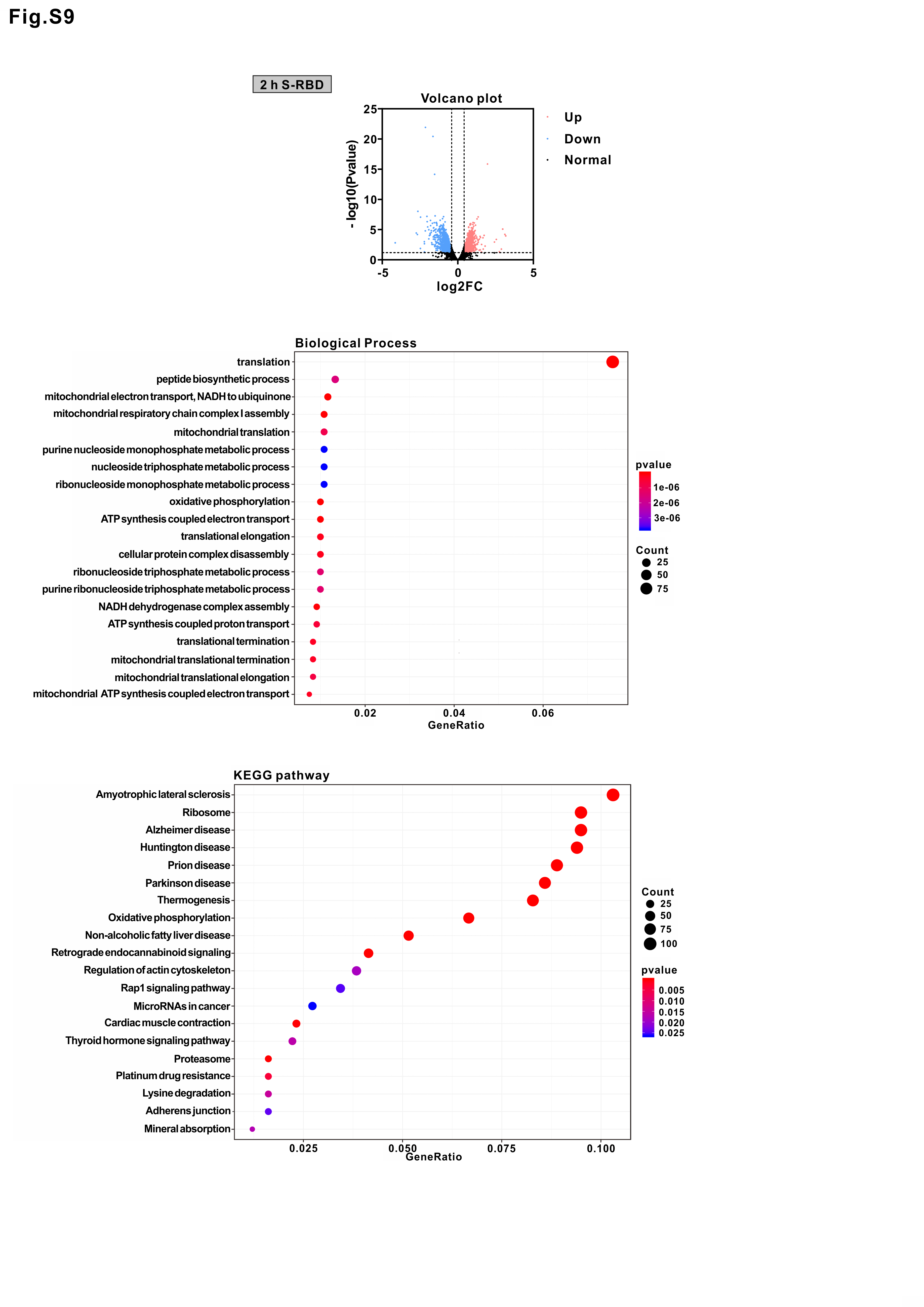


**Supplementary Figure 9: RNA-sequencing analysis showing the acute effects of prototypic strain S-RBD on human PAECs.**

Representing the volcano plots showing the differentially expressed genes, GO analysis of the biological process and KEGG pathway analysis for the differentially expressed genes induced by 2-hour treatment of prototypic strain S-RBD, normalized to IgG 2-hour, n = 3 in each group.


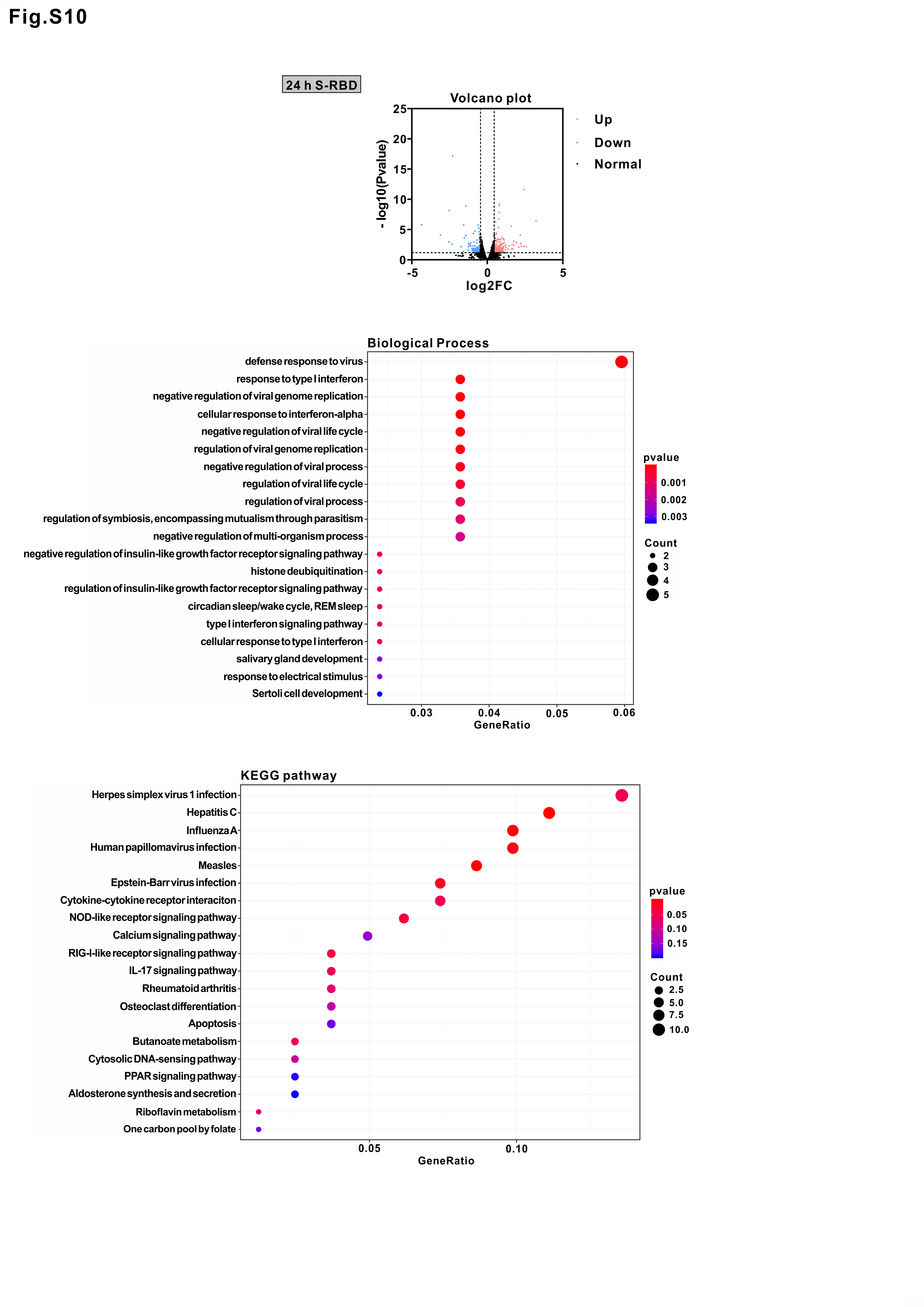


**Supplementary Figure 10: RNA-sequencing analysis showing the prolonged effects of prototypic strain S-RBD on human PAECs.**

Representing the volcano plots showing the differentially expressed genes, GO analysis of the biological process and KEGG pathway analysis for the differentially expressed genes induced by 24-hour treatment of prototypic strain S-RBD, normalized to IgG 24-hour, n = 3 in each group.


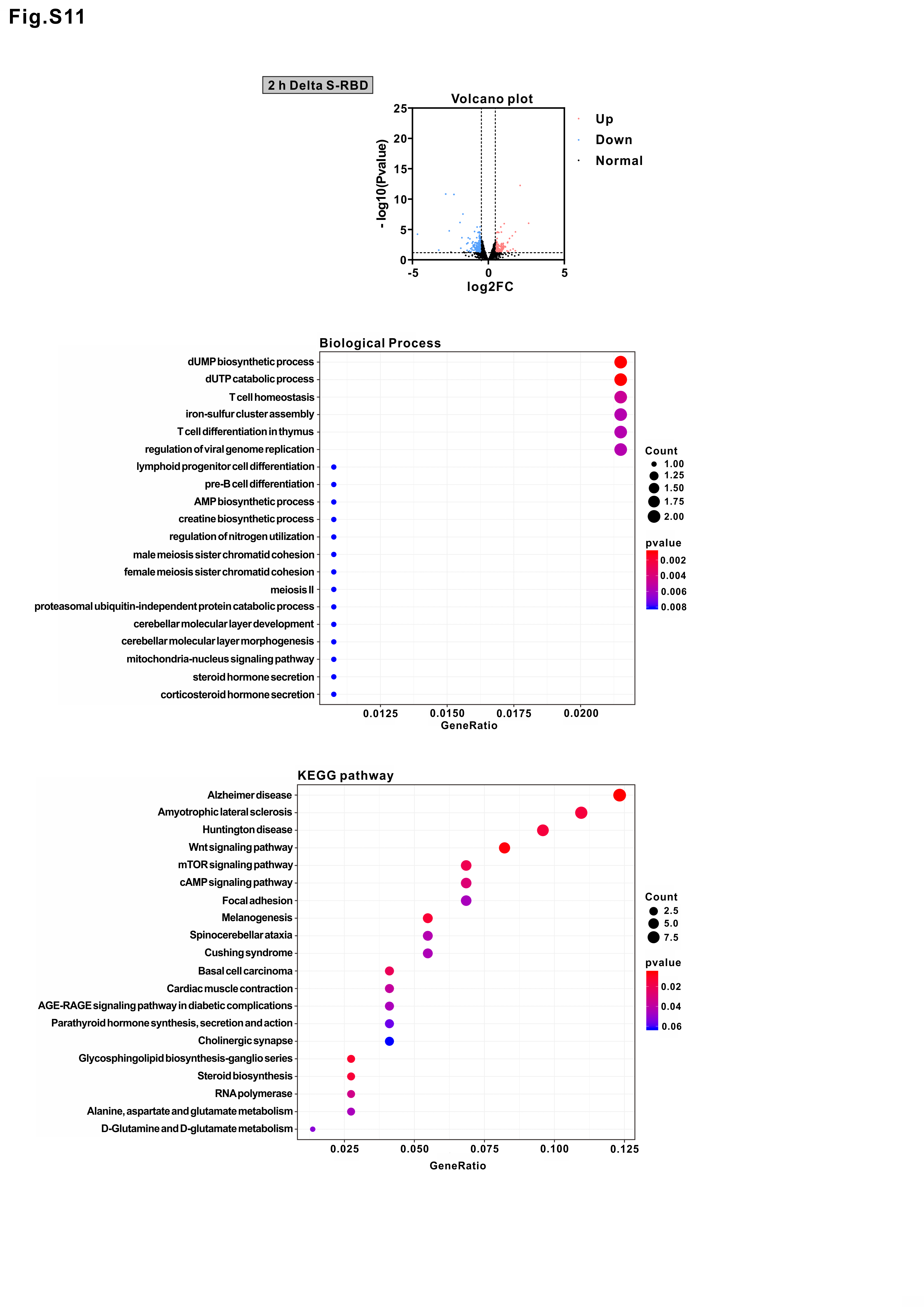


**Supplementary Figure 11: RNA-sequencing analysis showing the acute effects of Delta S-RBD on human PAECs.**

Representing the volcano plots showing the differentially expressed genes, GO analysis of the biological process and KEGG pathway analysis for the differentially expressed genes induced by 2-hour treatment of Delta S-RBD, normalized to IgG 2-hour, n = 3 in each group.


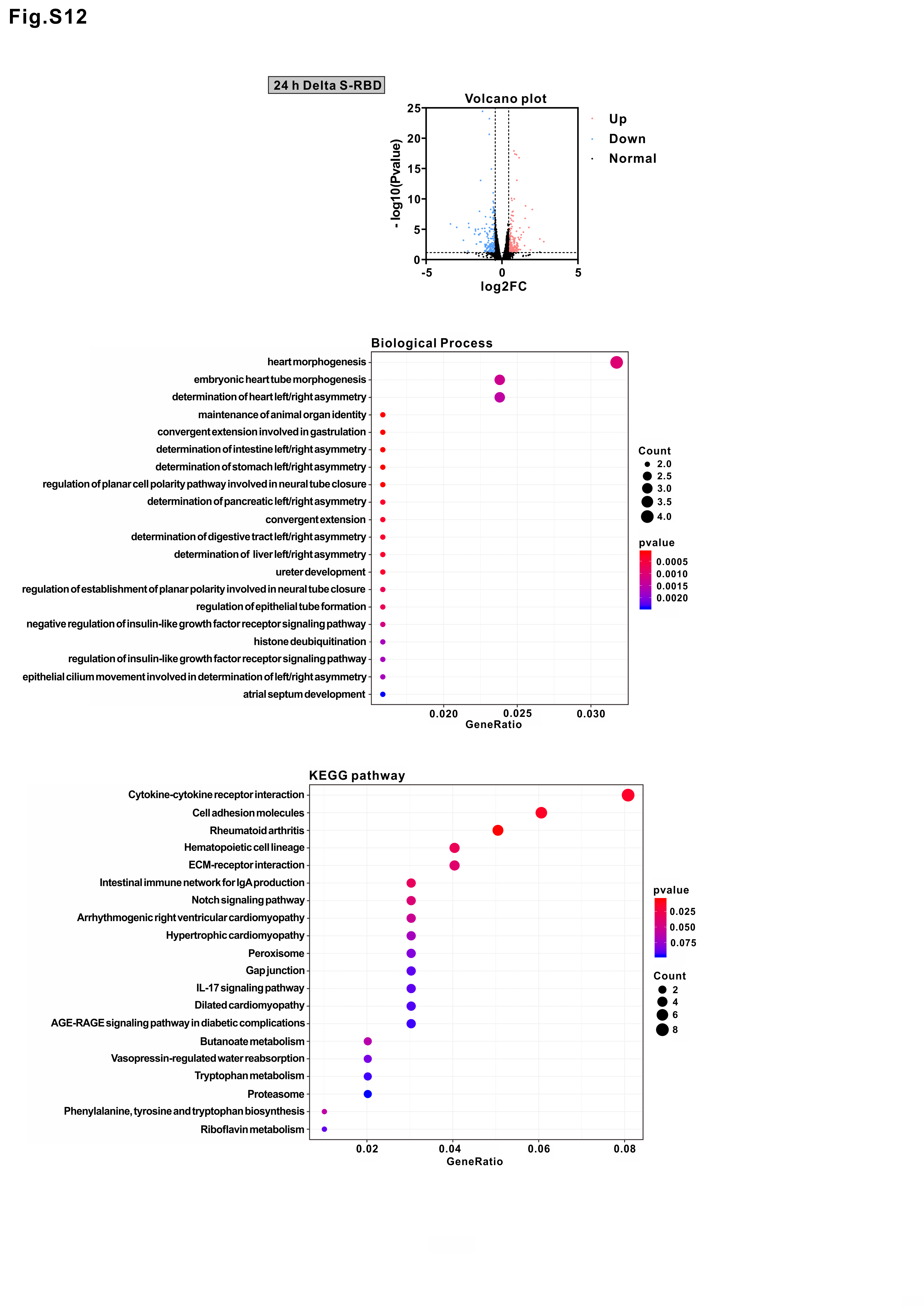


**Supplementary Figure 12: RNA-sequencing analysis showing the prolonged effects of Delta S-RBD on human PAECs.**

Representing the volcano plots showing the differentially expressed genes, GO analysis of the biological process and KEGG pathway analysis for the differentially expressed genes induced by 24-hour treatment of Delta S-RBD, normalized to IgG 24-hour, n = 3 in each group.


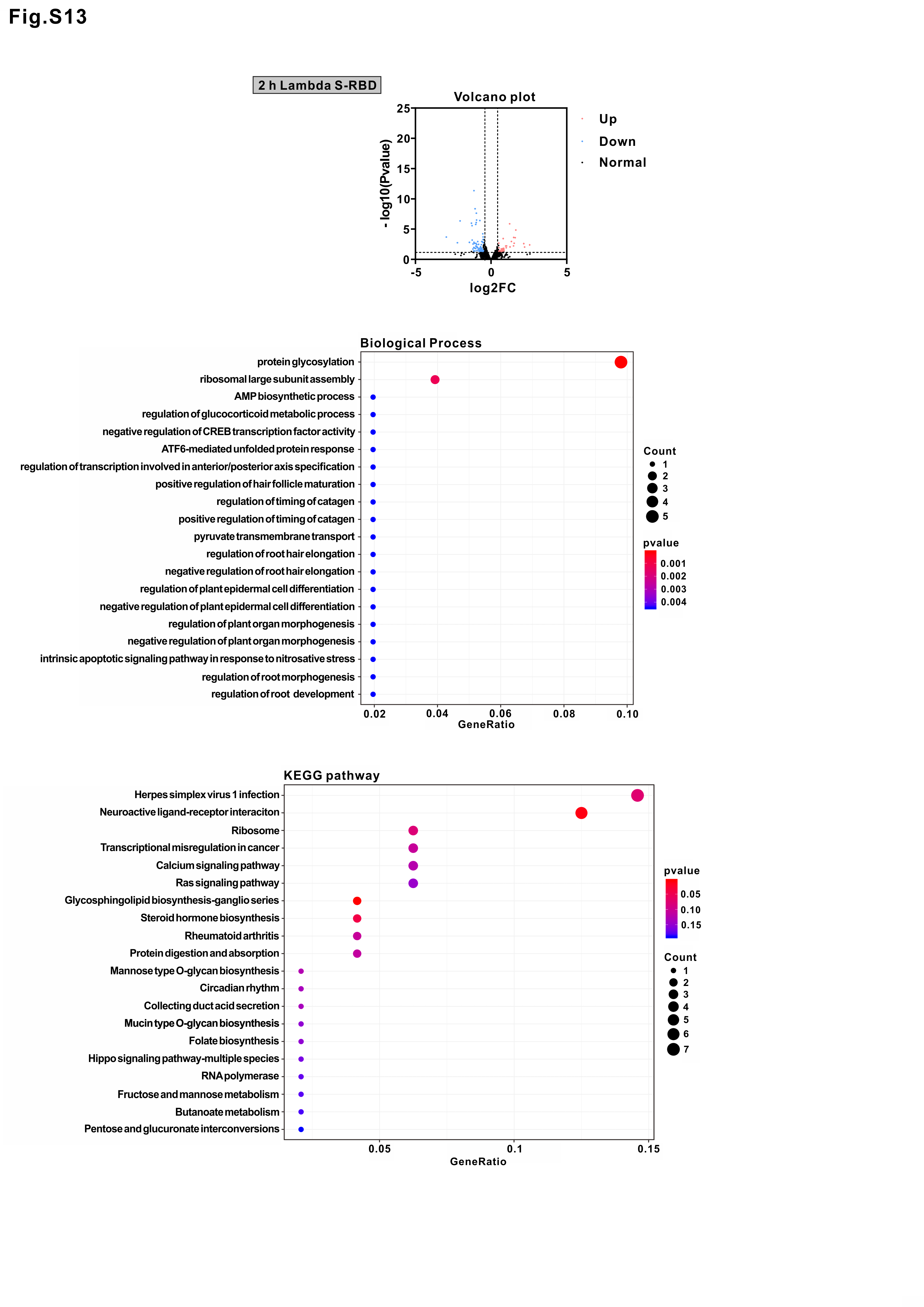


**Supplementary Figure 13: RNA-sequencing analysis showing the acute effects of Lambda S-RBD on human PAECs.**

Representing the volcano plots showing the differentially expressed genes, GO analysis of the biological process and KEGG pathway analysis for the differentially expressed genes induced by 2-hour treatment of Lambda S-RBD, normalized to IgG 2-hour, respectively, n = 3 in each group.


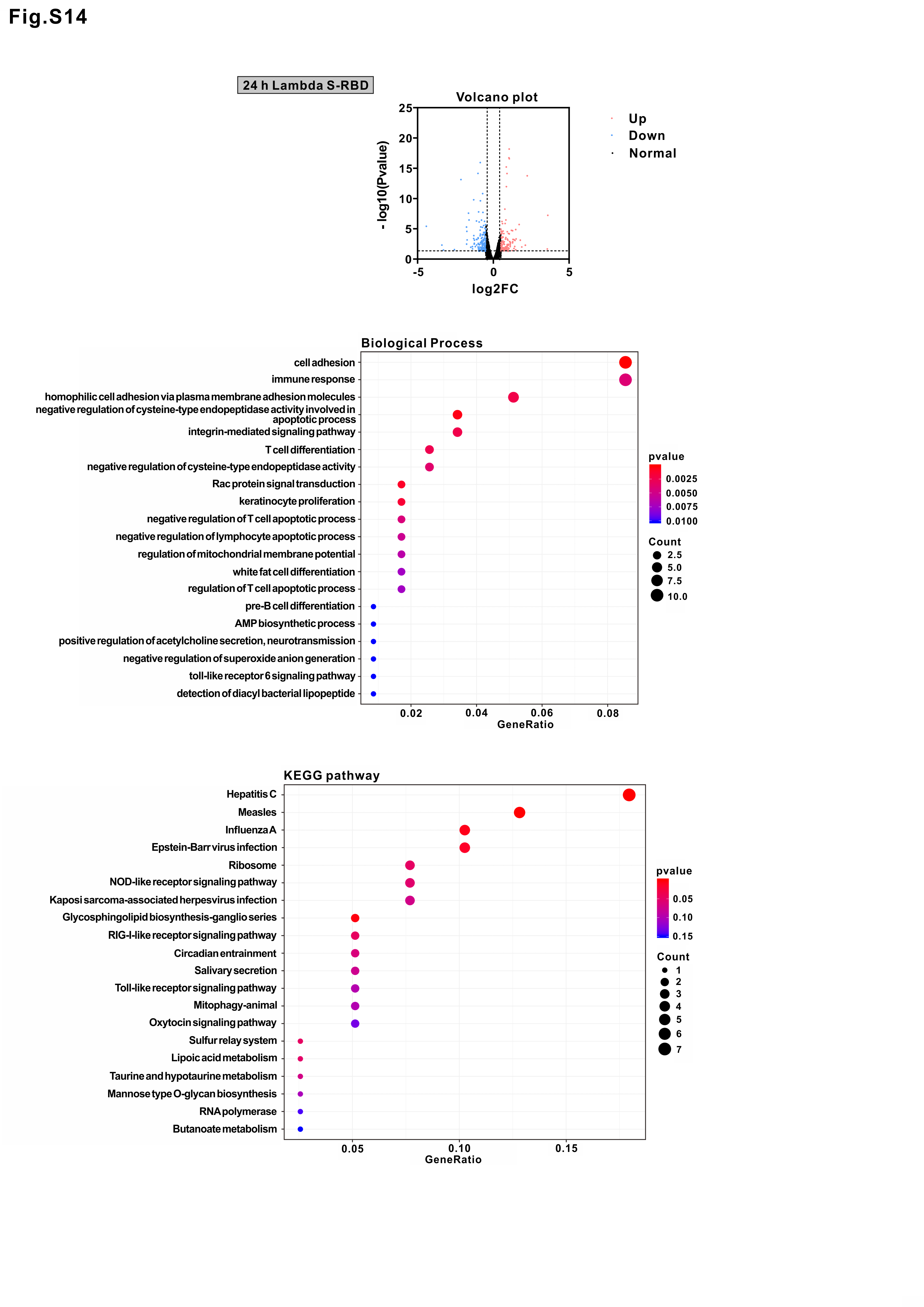


**Supplementary Figure 14: RNA-sequencing analysis showing the prolonged effects of Lambda S-RBD on human PAECs.**

Representing the volcano plots showing the differentially expressed genes, GO analysis of the biological process and KEGG pathway analysis for the differentially expressed genes induced by 24-hour treatment of Lambda S-RBD, normalized to IgG 24-hour, respectively, n = 3 in each group.
